# The unusual structure of the PiggyMac cysteine-rich domain reveals zinc finger diversity in PiggyBac-related transposases

**DOI:** 10.1186/s13100-021-00240-4

**Published:** 2021-04-29

**Authors:** Marc Guérineau, Luiza Bessa, Séverine Moriau, Ewen Lescop, François Bontems, Nathalie Mathy, Eric Guittet, Julien Bischerour, Mireille Bétermier, Nelly Morellet

**Affiliations:** 1 Université Paris-Saclay, CEA, CNRS, Institute for Integrative Biology of the Cell (I2BC), 1 Avenue de la Terrasse, 91198 Gif sur Yvette Cedex, France; 2 Université Paris-Saclay, CNRS, Institut de Chimie des Substances Naturelles, UPR 2301, 1 Avenue de la Terrasse, 91198 Gif sur Yvette Cedex, France; 3 Present addresses: Université Grenoble Alpes, CNRS, CEA, Institut de Biologie Structurale (IBS), 71 Avenue des Martyrs, 38000 Grenoble, France; 4 Reproduction et Développement des Plantes UMR 5667, Ecole Normale Supérieure de Lyon, 46 Allée d’Italie, 69364 Lyon Cedex 07, France

**Keywords:** Domesticated transposase, Zinc finger structure, Genome rearrangements, Ciliates, Histones

## Abstract

**Background:**

Transposons are mobile genetic elements that colonize genomes and drive their plasticity in all organisms. DNA transposon-encoded transposases bind to the ends of their cognate transposons and catalyze their movement. In some cases, exaptation of transposon genes has allowed novel cellular functions to emerge. The PiggyMac (Pgm) endonuclease of the ciliate *Paramecium tetraurelia* is a domesticated transposase from the PiggyBac family. It carries a core catalytic domain typical of PiggyBac-related transposases and a short cysteine-rich domain (CRD), flanked by N- and C-terminal extensions. During sexual processes Pgm catalyzes programmed genome rearrangements (PGR) that eliminate ~ 30% of germline DNA from the somatic genome at each generation. How Pgm recognizes its DNA cleavage sites in chromatin is unclear and the structure-function relationships of its different domains have remained elusive.

**Results:**

We provide insight into Pgm structure by determining the fold adopted by its CRD, an essential domain required for PGR. Using Nuclear Magnetic Resonance, we show that the Pgm CRD binds two Zn^2+^ ions and forms an unusual binuclear cross-brace zinc finger, with a circularly permutated treble-clef fold flanked by two flexible arms. The Pgm CRD structure clearly differs from that of several other PiggyBac-related transposases, among which is the well-studied PB transposase from *Trichoplusia ni*. Instead, the arrangement of cysteines and histidines in the primary sequence of the Pgm CRD resembles that of active transposases from *piggyBac*-like elements found in other species and of human PiggyBac-derived domesticated transposases. We show that, unlike the PB CRD, the Pgm CRD does not bind DNA. Instead, it interacts weakly with the N-terminus of histone H3, whatever its lysine methylation state.

**Conclusions:**

The present study points to the structural diversity of the CRD among transposases from the PiggyBac family and their domesticated derivatives, and highlights the diverse interactions this domain may establish with chromatin, from sequence-specific DNA binding to contacts with histone tails. Our data suggest that the Pgm CRD fold, whose unusual arrangement of cysteines and histidines is found in all PiggyBac-related domesticated transposases from *Paramecium* and *Tetrahymena*, was already present in the ancestral active transposase that gave rise to ciliate domesticated proteins.

**Supplementary Information:**

The online version contains supplementary material available at 10.1186/s13100-021-00240-4.

## Background

Transposons or transposable elements (TEs) are mobile genetic elements that have been shown to colonize the genome of organisms from all kingdoms of life [1]. TEs are divided into two major classes: class I TEs or retrotransposons, which use an RNA intermediate for their “copy-and-paste” transposition, and class II TEs, also called DNA transposons, which transpose through a DNA intermediate [2]. A sub-class of DNA transposons follow a “cut-and-paste” transposition mechanism, in which they are excised from their donor site before being inserted into a new target locus. Transposases are enzymes encoded by DNA transposons [3]. They generally bind to terminal inverted repeats (TIRs) at transposon ends and catalyze their movement from one genomic position to another. TEs can have harmful consequences when they invade coding or regulatory regions, inactivating or altering the regulation of host genes, but this may sometimes set up novel regulatory networks beneficial to the host [4]. The genome sequences of many species have also revealed a number of previously unrecognized TE-derived genes. In some cases, transposase genes have been coopted by their hosts during evolution to create new cellular genes, conferring an adaptive benefit to their host [5, 6]. This is called transposase “domestication”.

The *piggyBac* transposon that was originally isolated from *Trichoplusia ni* (*T. ni*) [7] is a well-studied DNA transposon with efficient transposition activity in many insect and mammalian species (reviewed in [8]). It inserts almost exclusively into TTAA target sites and restores the original TTAA sequence after excision, leaving no footprint at its donor site [9]. PB, the PiggyBac transposase encoded by the *T.ni piggyBac* transposon, catalyzes the DNA strand breakage and rejoining reactions that take place during transposition [10]. *PiggyBac*-like elements (PBLE), some of which were shown to be active, have been found in the genomes of numerous organisms including fungi, plants and a wide array of metazoans [11–13]. In addition, *piggyBac* transposable element-derived (PGBD) transposases are present in several eukaryotic species, including five in human, but their cellular function has remained unclear in normal tissues [13, 14]. The most ancient, Pgbd5, is active in transposition [15] and promotes genomic rearrangements in solid tumors [16], but no catalytic function has been attributed to the other four (Pgbd1 to Pgbd4) [17]. Intriguing instances of domesticated PGBD transposases playing an essential role during development were reported in the ciliates *Paramecium tetraurelia* (PiggyMac and its PiggyMac-like partners) [18, 19] and *Tetrahymena thermophila* (Tpb1, Tpb2, Tpb6 and Lia5) [20–23].

Ciliates are unicellular eukaryotes, in which two functionally distinct types of nuclei coexist in the same cytoplasm (reviewed in [24, 25]). The diploid micronucleus (MIC) transmits the germline genome to sexual progeny during reproduction. The highly polyploid somatic macronucleus (MAC), derived from the MIC and responsible for gene transcription, is destroyed at each sexual cycle. In the new developing MAC of *P. tetraurelia*, the genome undergoes amplification from 2n to 800n, while extensive programmed genome rearrangements take place. These consist in the precise elimination of ~ 45,000 single-copy, non-coding and short Internal Eliminated Sequences (IESs), representing ~ 3% of all germline DNA, and the heterogeneous removal of regions encompassing TEs and other DNA repeats, which altogether constitute ~ 25% of the germline genome [26, 27]. Because ~ 50% of *P. tetraurelia* genes are interrupted by at least one IES in the MIC, precise IES excision is essential for the assembly of functional genes in the new MAC. The conserved TA dinucleotides that flank each IES are targeted for DNA cleavage by the PiggyMac (Pgm) endonuclease associated with its PiggyMac-like (PgmL) partners [18, 19] and a single TA is retained at the excision site in the rearranged MAC genome. Developmentally regulated deposition of H3K9me3 and H3K27me3 heterochromatin marks by the Ezl1 histone methyltransferase is required for the elimination of all TEs and ~ 70% of IESs [28, 29]. Similarly, *T. thermophila* uses Tpb2 [20], a Pgm ortholog, to eliminate about a third of its germline genome through the heterochromatin-driven imprecise excision of ~ 12,000 TE-related IESs [30–33].

Pgm, a 1065-amino acid protein, plays an essential catalytic role in DNA cleavage at IES ends [34], while its PgmL partners are likely architectural subunits organizing the excision complex [19]. A comparison with the PB transposase indicates that Pgm is composed of four distinct domains [19, 34]: (i) a 220-amino acid N-terminal domain; (ii) a 424-amino acid core domain typical of transposases from the PiggyBac family, which includes the three conserved aspartic acids (D_401_D_491_D_609_) that are essential for IES excision; (iii) a short cysteine-rich domain (CRD) also found, with some variations in the number and order of its cysteine and histidine residues, in PBLE transposases and domesticated PGBD proteins from other organisms [13, 19]; and (iv) a predicted C-terminal coiled-coil structure (CC) encompassing the last 307 residues, which appears to be an innovation of ciliate domesticated PGBDs. Previously, we demonstrated that Pgm_ΔCRD_, a deletion mutant lacking the CRD, is unable to support IES excision during MAC development, showing that the CRD is essential for Pgm activity in vivo, although its exact role has remained unclear [34]. The PB CRD, essential for transposition, adopts a PHD-like cross-brace zinc finger fold (i.e. a zinc finger, in which the structural cores of the two zinc ions overlap) and binds specifically to a repeated DNA sequence motif present at *piggyBac* transposon ends [35]. Recent cryo-electron microscopy data indicated that the PB transposase assembles as a dimer within a synaptic complex composed of two *piggyBac* left ends, with the two PB CRDs binding together to a single end, introducing asymmetry within the complex [36]. *P. tetraurelia* IESs, intriguingly, do not carry a conserved motif that may serve as a sequence-specific recognition site for Pgm and, compared with PB, the primary sequence of the Pgm CRD exhibits a different arrangement of its potentially zinc-coordinating residues [19]. Taken together, these observations have raised two questions: whether the Pgm CRD adopts the same structural fold as the PB CRD and whether it also interacts with DNA.

In the present study, we have addressed the two issues through a structure/function analysis. We used nuclear magnetic resonance (NMR) spectroscopy to determine the structure of Pgm(692–768), which contains the CRD, and found that it binds two zinc ions with two distinct coordination modes (His-Cys_2_-His (ZF1) and Cys_4_ (ZF2)), in a cross-brace zinc finger motif quite unusual for a domain that was previously proposed to interact with chromatin [24]. Pgm(692–768) adopts a circularly permutated binuclear treble-clef fold [37], similar to the fold observed only in the C1 cross-brace zinc finger motif of protein kinase C (PKC) superfamily members. We further show that the Pgm CRD, unlike the PB CRD, does not bind DNA in vitro, but interacts weakly with the N-terminal tail of histone H3 (residues 1–19), independently of the methylation state of Lys9. Our work opens new perspectives on the structural and functional diversity of the CRD of PBLE transposases and their domesticated derivatives.

## Results

### Variability of the CRD of PB transposases and domesticated PGBD proteins

**Fig. 1:**
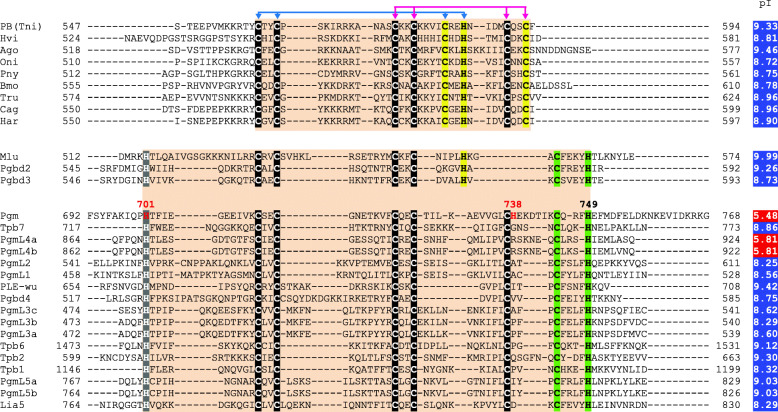
Sequence alignment of the CRDs of PBLE transposases and PGBD domesticated proteins. All accession numbers can be found in File S1. PBLE transposases: Ago (*Aphis gossypii*); Bmo (*Bombyx mori*); Cag (*Ctenoplusia agnate*); Har (*Helicoverpa armigera*); Hvi (*Heliothis virescens*); PB-Tni (*Trichoplusia ni*); Mlu (PiggyBat from *Myotis lucifugus*); PLE-wu (*Spodoptera frugiperda*). Domesticated PGBD transposases: Oni (*Oreochromis niloticus*); Pny (*Pundamilia nyererei*); Lia5, Tpb1, Tpb2, Tpb6 and Tpb7 (*Tetrahymena thermophila*); Pgm, PgmL1, PgmL2, PgmL3a/b/c, PgmL4a/b, PgmL5a/b (*Paramecium tetraurelia*); Tru (*Takifugu rubripes*); Pgbd2, Pgbd3 and Pgbd4 (*Homo sapiens*). In the sequence of Pgm(692–768), the coordinates of histidine residues are indicated, with His701 and His738 in red. In all sequences, the upstream histidine corresponding to Pgm His701 is highlighted in grey, when present. Conserved residues specific for the PB CRD are highlighted in yellow, those specific for the Pgm CRD are highlighted in green. For each peptide, isoelectric points (on the right: in blue or red for basic or acidic peptides, respectively) were calculated for the sequences highlighted in orange, using the ExPASy Compute pI/MW tool (https://web.expasy.org/compute_pi/)

Previous protein sequence alignments highlighted different Cys/His arrangements among the CRDs located C-terminal to the core domain of PBLE transposases and most PGBD domesticated transposases [13, 19]. The CRD of the *T. ni* PB transposase has a CxxC-CxxC-CxxH-CxxC motif (where C, H and x respectively denote cysteine, histidine and any other residue), also present in a subset of PBLE transposases and PGBD proteins (Fig. 1), which forms a PHD-like zinc finger coordinating two Zn^2+^ ions [35]. The putative variant motif (CxxC-CxxC-CH-CxxxH) found in the Pgm CRD was initially proposed to adopt a similar fold, in spite of the different number and position of its Cys and His residues [18]. However, primary sequence alignments have revealed that His738, adjacent to the fifth cysteine of the variant motif in Pgm, is not conserved in the CRDs of other ciliate PGBD domesticated transposases nor in PBLE transposases harboring an otherwise similar arrangement of their Cys and His residues (Fig. 1), calling into question the actual involvement of His738 in the folding of the Pgm CRD. We noticed instead that a histidine residue is systematically present at a variable distance upstream of the first conserved cysteine doublet in Pgm (His701) and other CRDs carrying the variant Cys/His arrangement. The observed differences in the primary sequence features of the Pgm and PB CRDs suggested that they might adopt different folds. This prompted us to solve the structure of the Pgm(692–768) variant domain.

### Zn^2+^ coordination mode of His701, His738 and His749

**Fig. 2:**
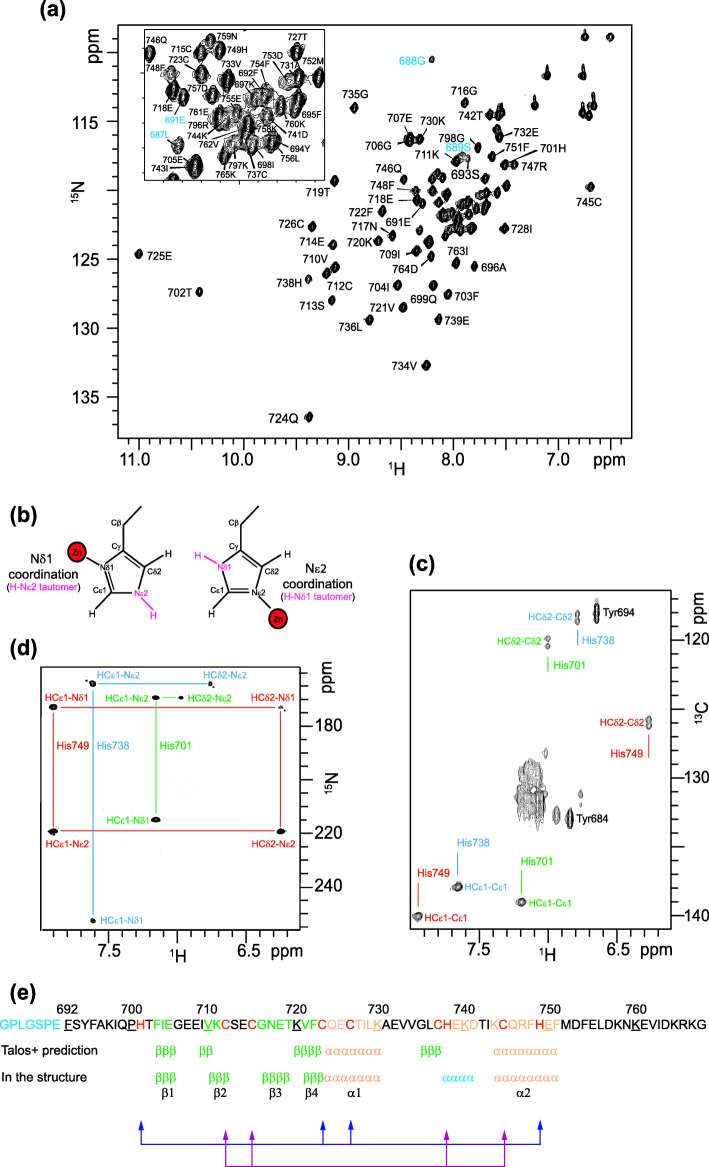
Determination of the histidine residues implicated in Zn^2+^ coordination. **a** ^1^H-^15^N HSQC spectrum of 250 μM ^15^N- and ^13^C-labeled Pgm(692–768)* at 800 MHz, in the presence of 5 mM Hepes pH 6.8, 25 mM NaCl at 20 °C. Pgm(692–768)* corresponds to residues 692–768 plus the first seven linker residues (in blue, see also panel e) left after PreScission cleavage of the GST-Pgm(692–768) fusion. **b** Schematic representation of the two tautomeric states of histidine able to complex zinc ions. The Nδ1 coordination form is associated to the H-Nε2 tautomeric form and the Nε2 coordination form is associated to the H-Nδ1 tautomeric form. Zinc ions are represented as red spheres. **c** ^1^H-^13^C HSQC (20 °C, pH 7.5) used to compare the Cδ2, Hδ2 and Cε1, Hε1 chemical shifts of His701, His738 and His749. **d** ^1^H-^15^N HSQC (20 °C, pH 7.5) used to compare the Nδ1 and Nε2 chemical shifts of His701, His738 and His749 **e** Sequence of the Pgm(692–768)* peptide used for NMR experiments. The Zn^2+^ ligands are highlighted in red. TALOS+ secondary structure predictions based on the experimental chemical shift information and those found in the 3D NMR structures of Pgm(692–768) are shown, with β-strands in green (703–705, 710–712, 718–719, 722–724) and α-helices in orange (725–730 and 745–751). The additional mini-helix observed in the final structures is highlighted in blue. The cross-brace arrangement of the two zinc-binding motifs is indicated by blue and pink arrows, each corresponding to one zinc-binding motif

The ^1^H-^15^N HSQC spectrum of Pgm(692–768)* obtained using NMR spectroscopy (Fig. 2a) exhibits marked dispersion of the ^1^H resonance chemical shifts with most cross-peaks being in the 6.6–11 ppm range, reflecting the presence of a well-folded structure. We found that zinc ions play a critical role in maintaining the structural integrity of Pgm(692–768)*. Indeed, in the presence of excess EDTA (10 mM) complexing zinc ions, the ^1^H 1D spectrum gives evidence of a random coil appearance, suggesting unfolding of the domain, since a significant decrease in the chemical shift dispersion was observed (Fig. S1). Histidine ligands can bind Zn^2+^ using two different coordination modes involving either of their endo-cyclic nitrogens (Nδ1 or Nε2). The Zn^2+^ coordination mode is related to the tautomeric form of histidine: when the deprotonated Nδ1 binds Zn^2+^, Nε2 is protonated and vice-versa (Fig. 2b). So, at first, we used NMR to determine the protonation states of histidine residues in Pgm(692–768)*. As shown previously [38], the Cδ2 and Cε1 chemical shifts of histidine can be used as a signature of the coordination mode of histidines in proteins. Using ^1^H-^13^C HSQC (Fig. 2c) we obtained the Cδ2 and Cε1 chemical shifts of His749 (126.0 ppm and 140.0 ppm respectively), which clearly indicate that His749 has an Nε2 coordination mode [38]. However the Cδ2 and Cε1 chemical shifts of His701 (120.2 ppm and 139.0 ppm, respectively) and His738 (118.4 ppm and 138.0 ppm, respectively) did not allow us to distinguish between coordinated and non-coordinated histidines. We could only conclude that His701 and His738 are in the H-Nε2 tautomeric form.

We also used long-range ^1^H-^15^N HSQC to determine the different forms of the three histidine residues of Pgm(692–768). The intensity of the cross-peaks (which depend on J-coupling) makes it possible to determine the chemical shift of Nε2 and Nδ1, which are inverted in the H-Nε2 tautomer compared to the H-Nδ1 tautomer. Whatever the tautomer, two strong correlations are observed between Nε2 and both H-Cε1 and H-Cδ2, and only one between Nδ1 and H-Cε1. The pattern observed for His701 and His738 has three connectivities (Hε1-Nδ1, Hε1-Nε2 and Hδ2-Nε2) (Fig. 2d), consistent with the formation of the H-Nε2 tautomer [39]. For His749, the observed pattern is constituted of four connectivities (HCε1-Nε2, HCδ2-Nε2, HCε1-Nδ1 and HCδ2-Nδ1) compatible with the formation of the H-Nδ1 tautomer (Fig. 2d). So His701 and His738 Nδ1 on the one hand and His749 Nε2 on the other are potentially available to interact with Zn^2+^. Comparison of the differences between the ^15^Nδ1 and ^15^Nε2 chemical shifts of His701 (45.7 ppm), His738 (88.4 ppm) and His749 (46.0 ppm), to that observed for a non-Zn^2+^-interacting histidine residue (about 80 ppm on average) allows us to propose that His701 and His749, but not His738, are coordinated to Zn^2+^.

Zn^2+^ coordination was further confirmed for His701 by atomic emission spectroscopy (Table S1). Mutating His701 to a serine residue caused a decrease in zinc emission compared to wild type Pgm(692–786). However mutation of His738 did not significantly affect zinc emission. Combining our NMR and atomic emission spectroscopy results, we can unambiguously discern that His701 Nδ1 and His749 Nε2 are involved in the coordination of Zn^2+^ while His738 is not.

### Structures of the Pgm(692–768) domain

A TALOS+ analysis [40] was performed to obtain estimations of the Pgm(692–768)* secondary structure and the phi and psi backbone torsion angles using the HN, Hα, Cα, Cβ, CO and N chemical shifts. The TALOS+ results reveal that Pgm(692–768)* is constituted of two α-helices encompassing residues 724–730 and 744–751 and four β-strands composed of residues 703–705, 709–710, 720–723 and 735–737 (Fig. 2e).

**Fig. 3:**
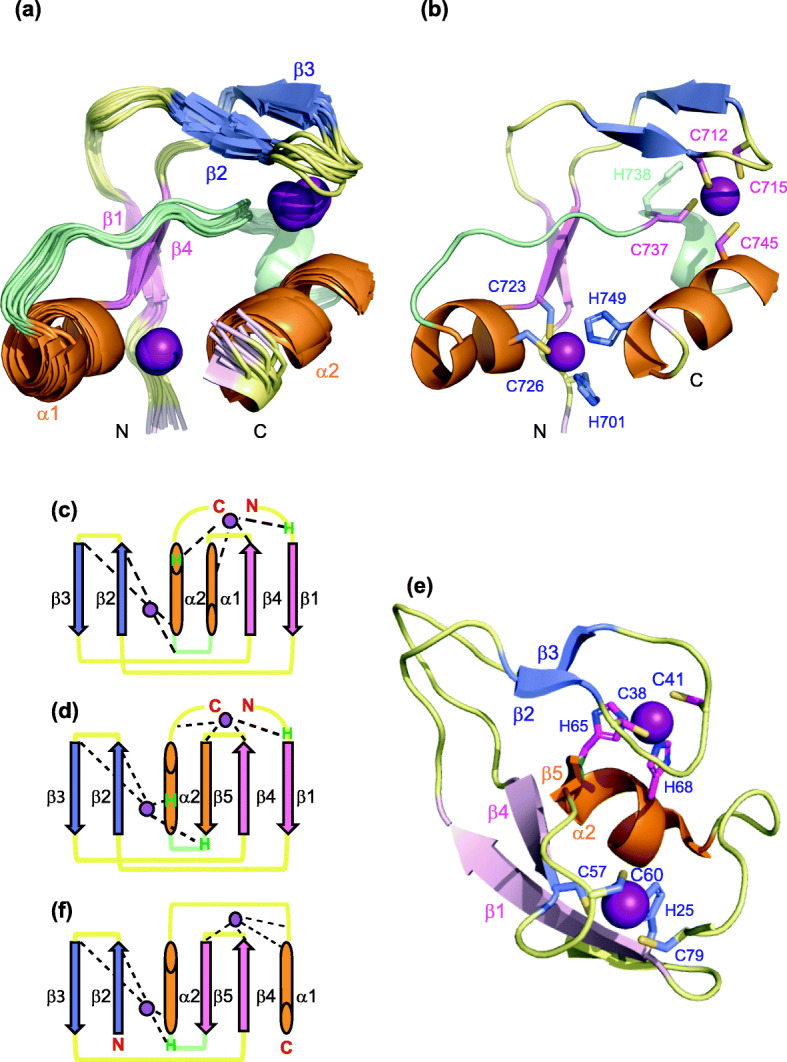
Structure of the Pgm CRD. **a** Backbone superposition on residues (701–751) of the best 15 structures generated with CYANA, with the two anti-parallel β-sheets in pink and blue, the two longest α-helices in orange (the secondary elements are highlighted according to the BMRB protein structure validation suite result report) and Zn^2+^ ions in purple. The same color code is used in all panels. **b** The cysteine and histidine residues involved in the coordination of Zn^2+^ ions are shown as sticks and colored by atom types: His701, Cys723, Cys726, His749 (blue) and Cys712, Cys715, Cys737, Cys745 (pink) are respectively involved in the formation of each of the two zinc-binding motifs. Atoms from His738, which is not implicated in Zn^2+^ coordination, are colored in green. **c** Topology diagram of Pgm(692–768). **d** Topology diagram of the C1 domain of Rho-associated protein kinase 2 (ROCK II) (PDB id 2ROW). **e** Structure of the ROCK II C1 domain. **f** Topology diagram of the typical PHD domain. In panels c, d and f, Zn^2+^ coordination is highlighted by dotted lines. Only Zn^2+^-coordinating histidine residues are indicated (H, in green), all others ligands are cysteine residues (not indicated). β-strands are represented as arrows, helices as cylinders and Zinc ions as purple spheres

CYANA structure calculations were performed using distance and angle restraints that imposed tetrahedral coordination of two Zn^2+^ ions: the first one with the His701 Nδ1, Cys723 S, Cys726 S and His749 Nε2 atoms and the other one with the Cys712 S, Cys715 S, Cys737 S and Cys745 S atoms. Fifteen structures were selected for structural analysis (Table 1) and are superimposed in Fig. 3a and S2. The structure of Pgm(692–768) reveals a well-defined globular domain for residues His701 to His749, with a root mean square deviation (rmsd) of 0.54 ± 0.19 Å from the average structure on the backbone atoms, and a cross-brace arrangement of the two zinc-binding motifs (Figs. 2e and 3b). The CRD structure is composed of two anti-parallel β-sheets and two α-helices, plus a mini helix composed of four residues (residues 738–741). The first β-sheet contains two strands, β1 (residues 703–705) and β4 (residues 721–723). The second β-sheet consists of two strands, β2 (residues 710–712) and β3 (716–719) and is part of a long bent hairpin extending from residues 706 to 720, positioned above the core formed by the two helices (Fig. 3a and b). A high degree of flexibility is observed for the N- and C-terminal regions (Fig. S2).

**Table 1:** NMR and refinement statistics for Pgm(692-768)* structures generated with CYANA

**Experimental restraints**	
Total number of restraints	1066
Intra-residual and sequential	736
Medium range (1 < |i**_**j| < 5)	108
Long range	222
TALOS+ Dihedral angles	116
**CYANA target function**	0.55 ± 0.09
**Rmsd from experimental restraints**^a^	
Upper limits *(*Å)	0.006 ± 0.003
Dihedral angles (deg)	0.392 ± 0.094
**Rmsd from idealized geometry**^a^	
Bonds *(*Å)	0.0012 ± 0.0008
Angles *(deg)*	0.53 ± 0.50
**Rmsd from mean structure**^a^	
Backbone atoms *(*Å)	0.54 ± 0.19 Å
Heavy atoms *(*Å)	1.03 ± 0.19 Å
**Ramachandran plot**^b^	
Most favored	90.0%
Additionally allowed	10.0%
Generously allowed	0.0%
Disallowed	0.0%

^a^Calculated over residues 701-749 of the 15 selected structures

^b^Calculated over ordered residues 698-714, 717-750, 762-764 of the 15 selected structures

### Analysis of ^15^N relaxation highlights chemical exchanges involving the N- and C-terminal regions of Pgm(692–768)

**Fig. 4:**
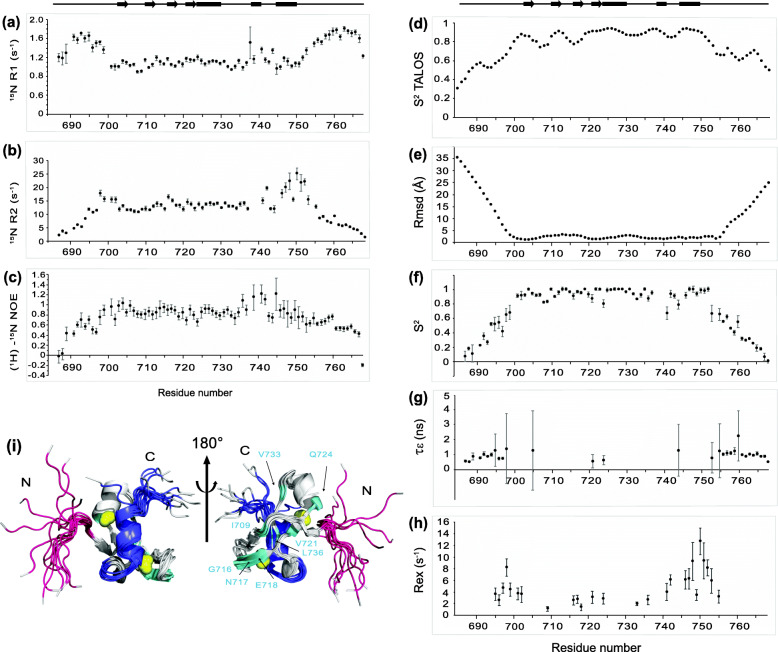
Relaxation rates and backbone dynamics of Pgm(692–768)*. Plots of the ^15^N R_1_ **a**, R_2_ **b** relaxation and heteronuclear {^1^H}-^15^N NOE **c** parameters obtained at 950 MHz ^1^H and 20 °C, using 0.5 mM of the ^13^C-^15^N-labeled Pgm(692–768)* domain, as a function of residue number and solution secondary structure. β-sheets are represented as full arrows and α-helices as full rectangles. **d** Backbone order parameter *S*^*2*^, derived from the *chemical shifts*, was generated by TALOS+ [40]. **e** Plot of the local backbone rmsd calculated using the CcpNmr software [41] on the 15 structures (aligned on residues 685–768), as a function of residue number and solution secondary structure. Dynamic parameters were extracted from the ^15^N relaxation data using the model-free formalism of Lipari-Szabo with an isotropic reorientation model: **f** amplitude of the picosecond (ps) to nanosecond (ns) time scale motion (S^2^), **g** internal correlation time (τ_ε_) and **h** exchange contributions on the microsecond (μs) to millisecond (ms) timescale (R_ex_). **i** The structure of the Pgm CRD highlights residues with conformational exchange: (695–702) in pink, (741–755) in dark blue and 8 amino acids from the structured region in light blue. Zinc ions are represented as yellow spheres

We observed heterogeneity in the line widths of the peaks in the ^1^H-^15^N HSQC, some of them showing a significantly weaker intensity (Fig. 2a). To determine if heterogeneity results from an exchange process between different conformations, we monitored the dynamics of the Pgm(692–768)* backbone using NMR relaxation measurements at 950 MHz ^1^H frequency and at 20 °C (Fig. 4). NMR ^15^N relaxation measurements are a powerful tool to characterize dynamic processes for proteins in solution, over a wide range of time scales. ^15^N relaxation results from the motion of the ^1^H-^15^N bond vector, which represents the combination of the global movements of the protein (diffusion of a rigid body) and the movements of internal vectors (atomic bonds). Fast motions on a picosecond-nanosecond (ps-ns) scale can be characterized by heteronuclear ^15^N longitudinal relaxation rate (R_1_), transverse relaxation rate (R_2_) and ^15^N-{^1^H} heteronuclear nuclear Overhauser effect (hetNOE) of amide group resonances. Chemical exchange mechanisms are involved in general movements on the microsecond-millisecond (μs-ms) scale and contribute to the R_2_ transverse relaxation rate. They can be described by measuring the excess contribution (R_ex_) to R_2_.

The results show that ^15^N relaxation rates are relatively homogeneous over the region encompassing residues 701 to 737, with averaged R_1_, R_2_*,* hetNOE values of 1.07 ± 0.03 s^− 1^, 13.00 ± 0.56 s^− 1^ and 0.85 ± 0.09, respectively (Fig. 4a-c). In contrast, the N- and C-terminal extremities (residues 687–700 and 752–768) show overall higher ^15^N R_1_ and lower ^15^N R_2_ and hetNOE values, indicative of an elevated mobility of the segments flanking the cross-brace zinc finger. The order parameter *S*^*2*^ describes the amplitude of the internal motion of an H-N bond within the frame of the structure and is close to 1 for rigid bonds and 0 for flexible bonds. The regions with increased flexibility are also highlighted by the backbone order parameter *S*^*2*^ estimated by TALOS+ [40] (and derived from the *chemical shifts*), since the average TALOS-predicted *S*^*2*^ values are 0.55 and 0.63 for residues 685–700 and 756–768 respectively, compared to 0.86 for residues 701–755 from the well-structured domain (Fig. 4d). This is in agreement with the rmsd per residue, which is particularly high for the N- and C-terminal flexible regions, with average values around 20 Å (residues 685–700) and 15 Å (residues 756–768), compared with an rmsd of 2.5 Å for residues 701–755 (Fig. 4e).

We noted that the Pgm regions encompassing residues corresponding to the flexible N-terminal domain (695–699) on one hand, and the 741–744 loop, the α2 helix (745–751) and several residues (752–755) from the flexible C-terminal domain on the other hand, showed higher R_2_ values than the average R_2_ calculated for the first three quarters of the structured part of the CRD (residues 701–737) (Fig. 4b). This may be due to the contribution of μs-ms conformational or chemical exchange. Model-free analysis [42, 43] of ^15^N NMR relaxation data provides information in terms of internal mobility and the time scale of molecular motions. Beyond the global molecular motion described by the correlation time (τc), the description of internal motions is reflected by the squared order parameter (S^2^), the internal correlation time (τε), and the chemical exchange contribution (R_ex_) to the transverse relaxation (R_2_). The overall correlation time (τc) estimated from the R_2_/R_1_ ratios (6.62 ± 0.04 ns), is consistent with Pgm(692–768)* being in a monomeric state [44]. The mean S^2^ (0.92 ± 0.03) calculated for residues 701–755 is consistent with a rigid and well structured domain (Fig. 4f). Residues from the N- and C-terminal regions showed a mean S^2^ of 0.38 ± 0.06 and 0.32 ± 0.03 respectively: internal correlation times (τ_ε_) corresponding to motions in the nanosecond time-scale had to be introduced for these residues to fit the relaxation data (Fig. 4g), consistent with the N- and C-terminal regions being very flexible. As a result of model-free analysis, significant chemical exchange contributions were introduced for two regions (residues 695–702 and 741–755) and for eight amino acids scattered inside the structured region (Fig. 4h). These exchanges may result from the long-range tertiary folding of the Pgm CRD, which brings the N- and C-terminal regions close to each other, allowing them to interact in a transient manner (Fig. 4i).

### No detectable binding of the Pgm CRD to DNA

**Fig. 5:**
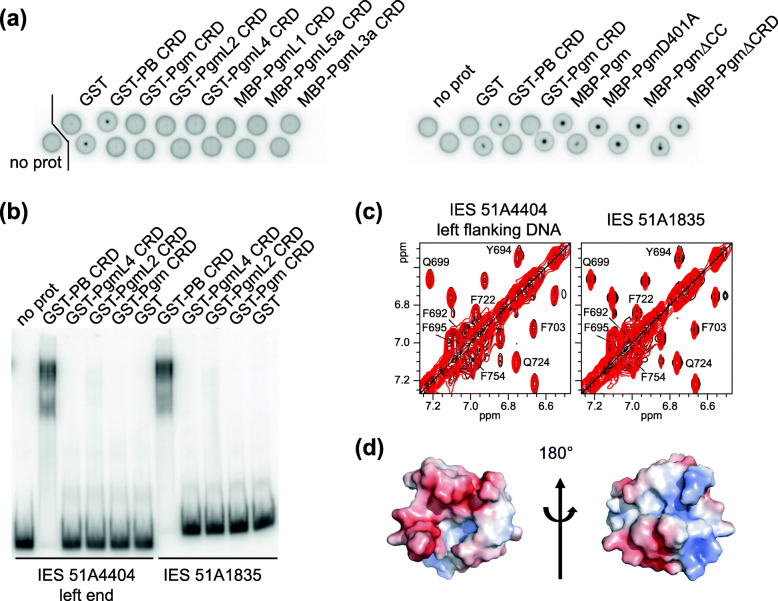
The Pgm and PgmL CRDs have no DNA binding activity. **a** DRaCALA assays using a radiolabeled 80-bp double-strand substrate carrying IES 51A1835 (28 bp) and purified GST or MBP N-terminal protein fusions. Loading onto the membrane was performed in duplicate for all complexes, except for the controls incubated without protein (no prot) or, on the left panel, with the GST tag alone, which were loaded only once. **b** EMSA assays were performed with a radiolabeled 70-bp double-strand substrate carrying 31 bp from the left end of IES 51A4404 (77 bp) and its left flanking MAC-destined sequences (left), or the same substrate as in panel a for IES 51A1835 (right). **c** NMR evidence for the lack of an interaction between the Pgm CRD and DNA. Aromatic regions of the ^1^H-^1^H NOESY spectra of Pgm(692–768) (black) are not perturbed in the presence of double-stranded DNA (red) from IES 1835 (left) or the left flanking region of IES 51A4404 (right). The sequences of the double-strand DNA substrates used in panels a, b and c are shown in Fig. S3. **d** Electrostatic potential calculated on one structure of Pgm(701–749). The red and blue colors in surface representations highlight negative and positive charges, respectively

We showed previously that the PB CRD is a double-strand DNA binding domain that specifically recognizes a conserved sequence motif (5′-TGCGT-3′/3′-ACGCA-5′) at the ends of the *piggyBac* transposon from *T. ni* [35]. To examine whether the Pgm CRD is also a DNA binding domain, we performed DRaCALA experiments (Differential Radial Capillary Action of Ligand Assay [45], see Materials and Methods), in which we compared the binding of purified GST-Pgm(692–768) and GST-PB(538–594) to a double-strand DNA carrying an IES, in the absence of competitor DNA to allow detection of non-specific DNA binding (Fig. 5a). We first chose IES *51A*1835 as a substrate (Fig. S3) because it is among the 30% of IESs, whose excision only depends upon the core Pgm/PgmL machinery and not on the deposition of epigenetic chromatin marks [46, 47]. We observed reproducible binding of GST-PB(538–594) to this DNA substrate, while no binding was detected in the absence of protein or following incubation with GST alone. Because IES *51A*1835 is unrelated to the natural specific substrate of the PB CRD, this confirms that the DRaCALA assay can reveal non-specific protein-DNA interactions. In contrast, we did not detect DNA binding with either Pgm(692–768) or the CRD of any PgmL protein. We found, however, that MBP fusions to full-length Pgm or its different variants – including MBP-Pgm_ΔCRD_ (in which the CRD was deleted), MBP-Pgm_ΔCC_ (carrying a deletion of the C-terminal extension) [34] and MBP-Pgm_D401A_ (in which the first aspartic acid of the catalytic triad was replaced by an alanine) – all bind DNA (Fig. 5a), indicating that the CRD is dispensable for the binding of full-length Pgm to DNA, even though it is essential for Pgm activity in vivo [34]. In parallel to DRaCALA, we performed Electrophoretic Mobility Shift Assays (EMSA) and detected no DNA binding activity for purified GST fusions with Pgm(692–768), PgmL2(540–614) or PgmL4(856–931), following incubation with double-strand DNA substrates carrying IES *51A*1835 or the left end of IES *51A*4404 (Fig. 5b and S3). We conclude from these experiments that the Pgm CRD has no DNA binding activity in vitro by itself.

To complete this analysis, we tested by NMR whether the Pgm CRD is capable of binding double-stranded DNA oligonucleotides carrying IES *51A*1835 and its left flanking sequences, or the MAC-destined sequence flanking IES *51A*4404 (Fig. S3). Cross-peaks corresponding to Pgm(692–768)* in ^1^H-^1^H NOESY spectra recorded in the absence and presence of either DNA displayed neither chemical shift nor intensity changes (Fig. 5c). This again stands in contrast to what we recently described for the PB CRD [35]. According to the electrostatic charge distribution calculated on one NMR structure of Pgm(692–768)*, the global surface charge of the Pgm cross-brace zinc finger (Pgm(701–749)) is negative (Fig. 5d), consistent with its calculated isoelectric point of 5.48 (Fig. 1). One face of the cross-brace zinc finger presents an even more negatively charged distribution than the other, suggesting that the two faces of Pgm(701–749) are accessible for electrostatic interactions with positively charged molecules, rather than with negatively charged molecules such as DNA.

Altogether, our results indicate that the Pgm CRD, unlike the PB CRD, does not bind DNA in vitro, in agreement with a globally negatively charged pattern. Of note, no DNA binding activity was detected for the CRDs of any PgmL protein, which all exhibit a similar arrangement of His and Cys residues relative to the Pgm CRD (Fig. 1).

### Interaction of the Pgm CRD with histone H3

**Fig. 6:**
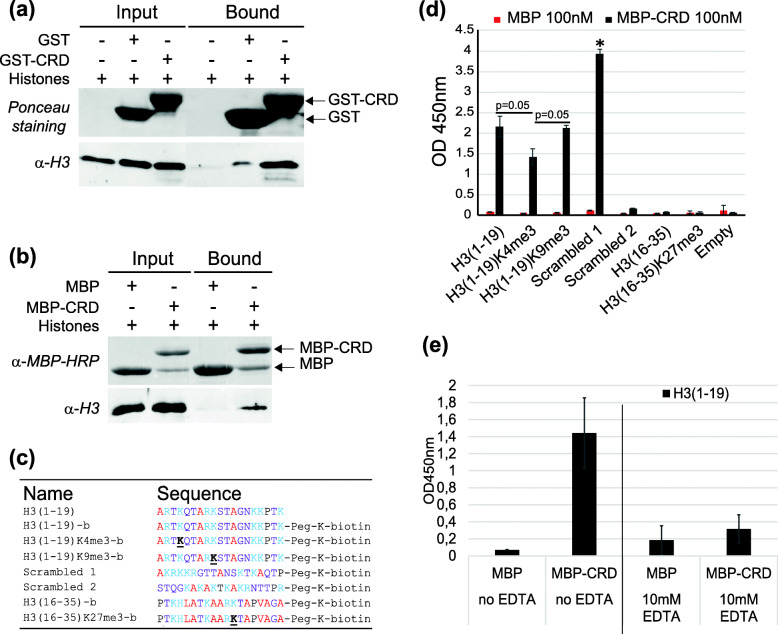
Interaction of the Pgm CRD with histone H3. **a** In vitro pulldown assay of *P. tetraurelia* histones with GST or GST-Pgm(692–768) (GST-CRD). **b** In vitro pulldown assay of *P. tetraurelia* histones with MBP or MBP-Pgm(692–768) (MBP-CRD). MBP fusion proteins and histone H3 were revealed on western blots using α-MBP-HRP and α-H3 antibodies, respectively (see Fig. S4 for the control of histone preparations and full-size blots with molecular weight markers). **c** Amino acid composition of the synthetic peptides used in this study. Methylated lysines are underlined. Hydrophobic residues are in red, residues with positively charged side chains in blue, residues with uncharged polar side chains in purple. C-terminally biotinylated peptides (−b) used in ELISA assays are indicated. **d** ELISA assays with 100 nM MBP or MBP-Pgm(692–768) (MBP-CRD) against unmodified H3(1–19), trimethylated H3(1–19)K4me3 and H3(1–19)K9m3, control peptides Scrambled 1 (*: TECAN absorbance detection at 450 nm close to saturation) and Scrambled 2, unmodified H3(16–35) and trimethylated H3(16–35)K27me3. *p*-values were calculated using the Wilcoxon-Mann-Whitney test calculator (https://ccb-compute2.cs.uni-saarland.de/wtest/); sample sizes m = 3, *n* = 3 and test variant H1: (H3(1–19)K4me3 signal) < (H3(1–19) signal) and H1: (H3(1–19)K4me3 signal) < (H3(1–19)K9me3 signal). **e** ELISA assays with MBP alone or MBP-Pgm(692–768) (MBP-CRD) against the H3(1–19) peptide. One hundred nM of each MBP fusion protein was loaded into H3(1–19)-coated wells either in absence (left panel) or in presence of 10 mM EDTA (right panel). Error bars represent the standard deviation between technical triplicates

In *P. tetraurelia*, the depletion of Ezl1, the histone methyltransferase responsible for the trimethylation of both H3K9 and H3K27 [29], inhibits the elimination of TEs and a large fraction of IESs [28]. This suggests that H3K9me3 and H3K27me3 modifications are involved in targeting the Pgm-associated complex to heterochromatin regions and drive their elimination. The primary sequence of the Pgm CRD shows a similar arrangement of its His and Cys residues compared with the CRD of Tpb2, the *Tetrahymena* orthologous domesticated transposase (Fig. 1) that was shown to interact with heterochromatin, in particular with the trimethylated N-terminal tail of histone H3 [48]. Since the Pgm CRD does not bind DNA, we examined whether it has histone-binding capability, similar to the Tpb2p CRD. We first performed in vitro pulldown assays of purified *P. tetraurelia* histones (Fig. S4) with GST-Pgm(692–768) and MBP-Pgm(692–768), and found that each fusion protein precipitates endogenous H3 (Fig. 6a-b). Little or no precipitation was detected with the GST or MBP tags alone, demonstrating that the interaction is mediated by Pgm(692–768) itself. We were not able to determine whether histones H2A/B or H4 were also precipitated, since no specific *Paramecium* histone antibodies were available.

To gain further insight into the involvement of histone N-terminal tail methylation in the interaction of the Pgm CRD with H3, we performed Enzyme-Linked ImmunoSorbent Assays (ELISA) using purified MBP-Pgm(692–768) and synthetic C-terminally biotinylated *Paramecium* H3(1–19) and H3(16–35) peptides. We used H3(1–19) peptides, either non-methylated or tri-methylated on Lys4 or 9, as well as two scrambled peptides (Scrambled 1 and Scrambled 2) containing the same amino acids as H3(1–19) in a different sequential order (Fig. 6c). We also used two H3(16–35) peptides: a non-methylated version and a modified variant carrying trimethylated Lys27. H3(1–19), H3(1–19)K4me3 and H3(1–19)K9me3 all gave an interaction signal with MBP-Pgm(692–768), while no signal was detected with MBP alone (Fig. 6d). Only a background signal was detected for H3(16–35) and H3(16–35)K27me3 in the presence of MBP-Pgm(692–768), indicating that the Pgm CRD does not interact with H3(16–35), whether Lys27 is methylated or not. Among all tested H3(1–19) peptides, unmethylated H3(1–19) and H3(1–19)K9me3 displayed the strongest interaction signals with MBP-Pgm(692–768), but interaction was also detected with H3(1–19)K4me3. This suggests that the Pgm CRD is able to interact with the N-terminal tail of histone H3 independently of the methylation state of Lys4 and 9. Similar interaction signals were obtained with all PgmL CRDs in the presence of the different H3 peptides (Fig. S5).

In order to monitor whether correct folding of the Pgm CRD is essential for its interaction with H3(1–19), we repeated the ELISA assay and incubated the MBP-Pgm(692–768) fusion with H3(1–19) in the presence of 10 mM EDTA, an excess concentration that triggers unfolding of the domain (Fig. S1). We observed a strong decrease of the interaction signal under these conditions (Fig. 6e), indicating that the cross-brace zinc finger fold of the Pgm CRD is essential for its interaction with H3(1–19). Surprisingly, we found that two mutant versions of the Pgm CRD still interact with H3, both in histone pulldown experiments and ELISA assays (Fig. S6): a single mutant carrying a C712S substitution in one of its zinc-coordinating motifs, which reduces the overall Zn^2+^ load to ~ 20% (Table S1), and a double mutant carrying the C712S and H701S substitutions expected to disrupt both zinc-coordinating motifs. Excess EDTA abolished the interaction signal with the double mutant (Fig. S6d), suggesting that the mutant CRD H701S + C712S still coordinates enough Zn^2+^ ions to maintain a structured fold and interact with H3(1–19).

### Analysis of the contacts between Pgm(692–768) and H3(1–19)

We observed that the two scrambled H3(1–19) peptides behaved differently in the presence of the Pgm CRD (Fig. 6d and S7a). Scrambled 1 gave an even greater interaction signal with MBP-Pgm(692–768) than H3(1–19), while only a background signal was observed with Scrambled 2. A comparison of peptide sequences highlights that, like H3(1–19), Scrambled 1 has an N-terminal alanine followed by five basic residues, while Scrambled 2 carries an uncharged STQ sequence at its N-terminus, suggesting the importance of a positive N-terminal charge for interacting with the Pgm CRD (Fig. 6c). The 30% reduced interaction signal of MBP-Pgm(692–768) with H3(1–19)K4me3 relative to unmodified H3(1–19) seems consistent with positive charges near the N-terminus of H3 being important for the interaction. Of note, a TOCSY experiment revealed chemical shift perturbation of the resonances of the first two residues of H3 (Ala1 and Arg2) in the presence of the Pgm CRD (Fig. S7b), confirming that the N-terminal residues of H3 are involved in the interaction.

**Fig. 7:**
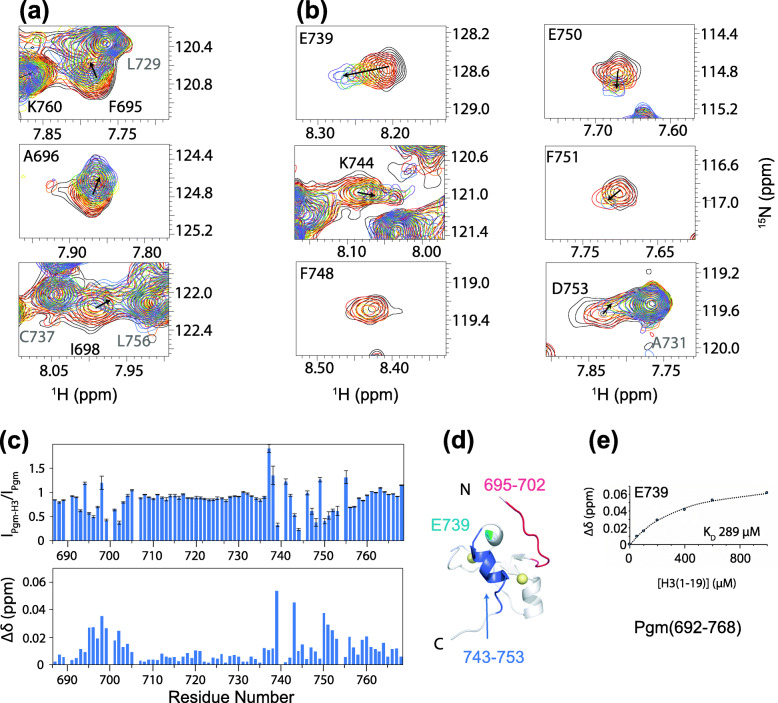
Pgm(692–768)* interacts with histone H3(1–19). **a** and **b** Superimposition of the ^1^H-^15^N HSQC spectra recorded at 800 MHz and 20 °C of 100 μM ^15^N-Pgm(692–768) in the absence (black) and presence of 0.5 (red), 1 (orange), 2 (yellow), 4 (green), 6 (blue) and 10 (purple) molar equivalents of H3(1–19). The panels show resonances from residues from the N-terminal **a** and the α2 helix **b** regions that display significant chemical shift perturbations and/or broadening upon addition of H3(1–19). The residues that do not undergo chemical shift variations are annotated in grey and the others in black. **c** Relative intensities and, below, chemical shift perturbations of the resonances of Pgm(692–768)* in the presence of a 10-fold excess of H3(1–19). **d** One of the ensemble structures of Pgm(692–768)* showing the most affected residues in the presence of H3(1–19) in dark blue and pink. These are located in two main regions: the flexible N-terminal extension (695–702) and the α2 helix (743–753), in addition to Glu739 (light blue) in the preceding loop. Zn^2+^ ions are represented as spheres. **e** Determination of a dissociation constant (K_D_) of 289 ± 70 μM using the chemical shift perturbations for residue E739

To further characterize the contacts between Pgm(692–768) and H3(1–19), we again used NMR spectroscopy. The effect of adding H3(1–19) to ^15^N-labeled Pgm(692–768)* was monitored by ^1^H-^15^N HSQC (Fig. 7a and b). We observed both differential broadening of a subset of signals and chemical shift perturbations in the presence of the histone peptide (Fig. 7c). Mapping of these perturbations highlighted two regions (Fig. 7d). The first one (695–702) is located within the flexible N-terminal extension upstream of the globular zinc finger structure (Fig. 7a). The second region (739–753) includes several acidic residues from the structured fold (Glu739 and Asp741 from the linker between helices α1 and α2 and Glu750 from helix α2 (743–753)) (Fig. 7b), with Glu739 displaying the largest chemical shift changes. Interestingly, these two regions in contact with the H3 peptide correspond to those for which we obtained evidence for conformational exchanges (Fig. 4f), possibly resulting from a transient interaction between each other (Fig. 7e). This experiment allowed us to calculate an apparent dissociation constant (K_D_) of 289 ± 70 μM (Fig. 7e), assuming a 1:1 binding, indicating that the interaction between the Pgm CRD and H3(1–19) is rather weak.

Taken together, our results indicate that the interaction of the Pgm CRD with H3(1–19) is weak, does not depend on Lys4 and Lys9 methylation and mostly relies on electrostatic interactions, with acidic residues of Pgm(692–768) contacting the positively charged N-terminus of H3.

## Discussion

### The Pgm CRD forms an unusual zinc finger structure

Programmed genome rearrangements in *P. tetraurelia* provide a nice illustration of the impact of transposons on their host genome plasticity and evolution. Pgm is an example of a catalytically functional domesticated transposase [18]. Yet little is known about the structure-function relationships of its different domains. In this study, we provide the first insight into Pgm structure and demonstrate that its CRD, previously shown to be essential for activity in vivo, binds two Zn^2+^, resulting in the formation of a cross-brace zinc finger. Additionally, we show that the Pgm CRD does not bind DNA but is capable of binding the N-terminal residues of histone H3 in vitro – although with no specific affinity for methylated lysines.

Zinc fingers are generally part of larger proteins known to play a role in almost all cellular processes. These domains, characterized either by their zinc-binding residues and their respective entanglement or by the fold of their secondary structure, have been classified into 40 families [37], among which are the most-populated and experimentally well-characterized Really Interesting New Gene (RING) [49, 50], Plant Homeo-Domain (PHD) [51] and FYVE families [52, 53]. Even if the RING, FYVE and PHD domains share a common structural fold and binding mode of two Zn^2+^ ions (Fig. 3f), they display significant diversity in the selection of their targets. PHD fingers [51], for instance, are found in known chromatin-modifying proteins and in many chromatin regulatory factors. They generally function as epigenetic effectors or readers that interact with modified or unmodified histone H3 tails [54, 55]. Upon interaction with a PHD domain, the H3 tail generally adopts an extended β strand-like conformation and aligns with the β sheet of the PHD zinc finger as a third antiparallel β strand (Fig. S8) [56]. We show here that the Pgm CRD does not fold into any of the canonical RING, FYVE or PHD structures, contrary to what we observed for the PB CRD [35]. It adopts a topology analogous to the C1 cross-brace zinc finger motif, which is highly conserved among the protein kinase C (PKC) superfamily members that share a common requirement for phospholipids for their kinase activity [57], however with a different number of β strands and α helices (Fig. 3d-e and S9). Despite this similar fold, the long accessible bent hairpin of Pgm(701–749) does not exhibit the hydrophobic pocket found in a typical C1 domain targeted by DAG/phorbol ester (Fig. S10).

### The Pgm and PB CRDs interact with different substrates

We observed that full-length Pgm binds DNA, as expected for an active endonuclease, whose catalytic site is responsible for the cleavage of IES boundaries (Fig. 5). However, contrary to what we reported for the PB CRD [35], we detected no DNA binding activity for the Pgm CRD in DRaCALA, EMSA or NMR assays, nor did we find DNA binding activity of the CRDs of its PgmL partners. The lack of DNA binding activity of the Pgm CRD may reflect important differences between cut-and-paste transposition and Pgm-mediated IES elimination. Indeed, even though *Paramecium* IESs are, at least for a large fraction, remnants of ancestral *Tc1/mariner* transposons [26], their excision is mediated by PiggyBac-related domesticated transposases, which belong to a distinct family of class II transposons [18, 19]. In addition, IESs carry no conserved motif that may serve as a specific recognition sequence for the excision complex. Available experimental evidence indicates that IES recognition is maternally controlled through the combined action of non-coding RNAs and epigenetic chromatin marks [58]. Our results suggest that, in contrast to the PB transposase, what drives Pgm to *Paramecium* IESs is not the direct binding of its CRD to IES DNA.

We observed instead that the Pgm CRD interacts with histone H3 with a weak binding affinity. The histone- rather than DNA-binding activity of the Pgm CRD is consistent with the charge distribution on the surface of the folded domain, which is mainly negatively charged (Fig. 5). In comparison, the PB CRD, which does bind DNA, is globally positively charged [35]. We mapped the interaction to two distinct regions of the Pgm CRD: one mainly hydrophobic region of the flanking N-terminal part (residues 695–702) and a second region (743–753) harboring acidic residues and corresponding approximately to the last α-helix (Figs. 4 and 7). When the cross-brace zinc finger fold is disrupted following addition of excess EDTA, these two regions may not properly fold together, leading to a strong reduction of the contact with H3 (Fig. 6). Of note, the Pgm CRD harbors two β-sheets that are potentially accessible for an interaction with the N-terminal tail of H3 in an extended β strand-like conformation, as observed for PHD domains, but they do not appear to take part in the interaction with H3(1–19) (Fig. S8).

For the histone H3 tail, several lines of evidence have pointed to positive charges at the N-terminus of H3 being involved in contacting the Pgm CRD (Fig. 6 and S7). The lack of specificity for tri-methylated Lys9 and Lys27 was somewhat unexpected, given the essential role of the histone methyltransferase Ezl1, which catalyzes both H3K9 and H3K27 tri-methylation, during programmed DNA elimination in *P. tetraurelia* [28, 29]. It was hypothesized previously that H3K9me3 and/or H3K27me3 drive the Pgm complex to Ezl1-dependent IESs and other heterochromatin-associated regions and target their elimination. We show here that the Pgm CRD interacts with H3(1–19) independently of Lys9 methylation and does not interact with the region encompassing Lys27. Taken together, our results rather suggest that the observed weak interaction of the Pgm CRD with the N-terminal tail of H3 is mediated through electrostatic charges and that we may not have identified a specific substrate of this domain. Another component of the Pgm-associated complex, which remains to be identified, may carry a specific H3K9me3 and/or H3K27me3 recognition domain and position the excision complex at Ezl1-dependent IES excision sites.

### Structure-function variability of the CRDs of PBLE transposases and domesticated PGBD proteins

We recently identified five groups of paralogous Pgm-like (PgmL) proteins in *P. tetraurelia* (PgmL1 – PgmL5) [19], none of which contains an intact DD(D/E) active site, suggesting that PgmLs are not catalytically active. All PgmLs carry a CRD, in which the zinc-binding Cys/His are conserved with respect to Pgm, suggesting that the PgmL CRDs may share the same tertiary fold in spite of otherwise divergent primary sequences (Fig. 1). Each PgmL is capable of forming complexes with Pgm, plays an essential role in IES excision and is required for the correct completion of autogamy. Due to their variant primary sequences, the PgmL CRDs have very different isoelectric points, with only the PgmL4 CRD exhibiting an acidic isoelectric point close to that of the Pgm CRD (Fig. 1). Despite these differences, all PgmL CRDs present comparable interaction signals with unmethylated or methylated H3(1–19) peptides in ELISA assays, while they interact neither with H3(16–35) nor H3(16–35)K27me3 (Fig. S5). We conclude that the Pgm and PgmL CRDs share similar binding properties to the N-terminal tail of H3, independently of the methylation state of Lys9 and 27. Future studies should address whether cooperative interaction between Pgm and PgmL CRDs may provide higher affinity and/or specificity to the recognition of histone tails by the Pgm-associated complex.

In the distantly related ciliate *T. thermophila,* the Tpb2 domesticated transposase catalyzes heterochromatin-driven DNA elimination during sexual reproduction [20]. The Tpb2 CRD is capable of binding heterochromatin and interacts with the N-terminal tail of histone H3 with a preference for tri-methylated proteins on Lys9 or Lys27 [48]. The fold of the Tpb2p CRD has not been characterized and seven histidine and cysteine residues were previously proposed to participate in the folding of a PHD zinc finger [48]. Based on sequence alignments (Fig. 1), we show here that the primary sequences of the Tpb2 and Pgm CRDs have a similar organization, with one histidine and seven cysteine residues, suggesting that both CRDs may adopt a similar cross-brace zinc finger structure. The weak interaction of the Pgm CRD with the N-terminal tail of H3 appears to require the coordination of Zn^2+^ ions involved in folding of the CRD (Fig. 6). Likewise, a mutant Tpb2 CRD with two of its potentially Zn^2+^-binding cysteine/histidine residues changed to alanine loses its ability to bind H3 N-terminal peptides in pulldown assays [48]. As a result, the full-length mutant Tpb2 fails to trigger heterochromatin body formation and is inactive for DNA elimination in vivo, demonstrating the importance of the Tpb2 putative fold in interacting with heterochromatin [48]. In spite of their structural similarity, the Pgm and Tpb2 CRDs have different isoelectric points and abilities to discriminate between methylated and non-methylated histone tails, which may reflect major differences in the mechanism and/or control of programmed DNA elimination between the two ciliates. Indeed, *Paramecium* IESs are all excised precisely from genes and non-coding regions, while only a subset depend upon H3 trimethylation to be excised. In contrast, all *Tetrahymena* IESs, which are all intergenic, require heterochromatin formation for their imprecise elimination.

PBLEs are found in numerous organisms, such as fungi, plants, insects, fishes and mammals [13] and some of them are active in transposition [11, 12, 59, 60]. A large-scale survey of PBLE transposons allowed the definition of four structural groups, based on the differential organization of DNA repeats at their ends [13]. In particular, the *piggyBat* transposon from the bat *Myotis lucifugus*, which belongs to the first group, carries simple 15-bp TIRs at its ends [11], while *piggyBac* from *T. ni* and *PLE-wu* from the insect *Spodoptera frugipeda* [12], representatives of the fourth group, carry complex TIRs with multiple internal repeats and may require complex interactions for the correct conformation of their transpososome [36]. While all PBLE transposases harbor a characteristic conserved domain (PF13843, or DDE_Tnp_1_7), which includes their catalytic site, the study by Bouallègue et al. [13] has highlighted the diversity of the Cys/His motifs found in their C-terminal CRDs. Our previous [35] and present studies indicate that different Cys/His motifs may adopt distinct structural folds. Sequence alignment of two active PBLE transposases (PLE-wu [12] and PiggyBat-Mlu [11]) shows that they share a conserved His (aligned with Pgm His701) followed by seven Cys or His residues (Fig. 1), suggesting that their CRDs may adopt the Pgm CRD fold. Thus, the variant fold of the Pgm CRD, also possibly found in other ciliate domesticated transposases (PgmL or Tpb proteins), may have been inherited from an ancestral active ciliate PBLE transposase quite distinct from the *T. ni* PB transposase, rather than evolved during the domestication process. In human, while Pgbd1 and 5 do not carry a recognizable CRD, Pgbd2, 3 and 4 exhibit a Pgm-like arrangement of His and Cys residues in the primary sequence of their CRDs (Fig. 1), again suggesting that they may have originated from active PBLE transposases carrying the Pgm CRD fold. Currently available data reveal no straightforward correlation between the DNA sequence organization at PBLE transposon ends and the putative fold of the CRDs of their respective transposases. Even within the fourth group of PBLEs, for instance, the PLE-wu CRD may adopt a similar structure to the Pgm CRD fold (Fig. 1). Whether the PLE-wu CRD, like the PB CRD, binds sequence-specifically to DNA repeats or associates with histones has not been established. More generally, the presence of similar structures does not allow us to predict the preferred substrate of the CRDs of PBLE transposases or their domesticated PGBD counterparts, emphasizing the versatility of these domains and the diversity of the interactions in which they may be involved.

## Materials and methods

### Sequence analysis

Protein sequence alignment was performed using MUSCLE 3.8 (https://www.ebi.ac.uk/Tools/msa/muscle/) and adjusted manually.

### Peptides

Untagged and biotinylated synthetic *Paramecium tetraurelia* histone H3 peptides (Fig. 6c) were synthesized by Proteogenix (Schiltigheim, France) and used without further purification.

### Expression and purification of GST-tagged CRDs

Synthetic DNA fragments (Integrated DNA Technologies) encoding Pgm(692–768) and its C712S and H701S + C712S mutant derivatives, PB(538–594), PgmL1(463–539), PgmL2(540–614), PgmL3a(471–550), PgmL4a(856–931) and PgmL5a(761–840) were PCR amplified and cloned between the *Eco*RI and *Xho*I restriction sites of plasmid pGEX6p1 (resulting plasmid sequences in File S2).

For NMR spectroscopy, BL21-Gold (DE3) *E. coli* cells expressing GST-Pgm(692–768) were grown at 37 °C in M9 minimal medium with ^15^NH_4_Cl (1 g/L) (Cambridge Isotope Laboratories, USA) and either D-glucose-^13^C_6_ (Cambridge Isotope Laboratories, USA) or unlabeled D-glucose (3 g/L) for expression of ^13^C/^15^N- or ^15^N-labeled Pgm CRD, respectively. At OD_600_ = 0.6 to 0.8, cells were induced with 0.2 mM Isopropyl β-D-1-thiogalactopyranoside (IPTG) for 3 h30 in the presence of 1 mM ZnSO_4_ and were then collected and suspended in buffer A (25 mM Tris–HCl pH 7.5, 0.15 M NaCl, 10% glycerol, 10 mM 2-Mercaptoethanol) supplemented with 0.5 mM phenylmethane sulfonyl fluoride (PMSF). Cells were lysed with a French press and the cleared supernatant was filtered through a 0.45 μm syringe filter before loading onto 2.5 ml of Glutathion Sepharose™ 4B resin (GE Healthcare) and incubation for 1 h at room temperature. The resin was washed five times with 30 mL of buffer A. The Pgm (692–768)* CRD peptide (Fig. 2e) was cleaved from the GST-tag using 100 units of PreScission protease overnight at 4 °C in 10 mL of buffer A. The supernatant was diluted 3-fold in 25 mM HEPES pH 7.6, 5% glycerol buffer (degassed solution under vacuum) and loaded onto a 1-mL HiTrap™ Q HP column (GE Healthcare). The CRD peptide was eluted through a linear gradient of 50 to 1000 mM NaCl in 25 mM HEPES pH 7.6, 5% glycerol buffer (25 column volumes). CRD-containing samples were identified by SDS-PAGE then pooled and dialyzed twice against NMR solution (5 mM (if ^15^N-labeled) or 0.5 mM (if unlabeled) HEPES pH 7.5, 25 mM NaCl) using a Spectra/Por3 dialysis membrane, prior to storage at − 80 °C. The whole purification procedure is summarized in Fig. S11.

For DNA or histone binding assays, BL21-Gold (DE3) *E. coli* cells expressing GST-CRD fusions or GST alone were grown at 37 °C in LB medium supplemented with 0.1 mM ZnSO_4_. Exponentially growing bacteria expressing each GST fusion protein were induced overnight at 16 °C in the presence of 0.1 mM IPTG or at 30 °C in the presence of 0.5 mM IPTG, then collected and suspended in buffer B (10 mM Tris pH 7.4; 500 mM NaCl; 100 μM ZnSO_4_; 1 mM DTT; 1% Triton + 1 cOmplete™ tablet EDTA-Free Roche EasyPack). Cell lysis was performed using sonication with a BioRuptor (Diagenode). Five hundred μl slurry glutathione sepharose beads 4B (GE healthcare) were pre-equilibrated 3 times in 2.5 ml of buffer B. Cleared lysates (10 mL) were loaded onto the beads and incubated overnight at 4 °C on a rotating wheel. After incubation, the beads were washed 4 times with 2.5 mL of buffer B (last wash on wheel for 10 min at 4 °C). Proteins were eluted twice with 500 μl 10 mM Tris pH 8 and 20 mM glutathione for 15 min at room temperature. Purified protein concentration was assessed with BiCinchoninic acid Assay (BCA kit ThermoFisher). The whole purification procedure is summarized in Fig. S11.

### Expression and purification of MBP fusion proteins

Synthetic DNA fragments (Integrated DNA Technologies) encoding MBP-PgmL1(463–539), MBP-PgmL2(540–614), MBP-PgmL3a(471–550), MBP-PgmL4a(856–931), MBP-PgmL5a(761–840) and the wild-type and mutant versions of MBP-Pgm(692–768), were PCR-amplified and inserted between the *Eco*RI and *Pst*I sites of the pMAL-c2X vector (New England Biolabs, see plasmid sequences in File S2). Expression and purification of MBP-CRD fusions in BL21-Gold (DE3) *E. coli* cells was performed as described above for GST-CRD fusions, except that each cleared supernatant was loaded onto a 1-ml MBP-Trap HP Prepacked Column (GE-Healthcare) for affinity purification and the MBP-CRD was eluted with 10 mM maltose. All purified preparations are shown in Fig. S11. For those preparations, in which some of the free MBP tag was released upon over-expression or during bacterial lysis, the fraction of the full-length protein was estimated from the ratio of band intensities obtained from Instant blue-stained gels or from western blots. Band intensities were quantified using the Imagelab software (BIORAD). Only the amount of full-length fusion protein was taken into account to calculate the amount of input CRD in histone-binding assays.

For the expression of MBP-PgmD401A, the GAC codon encoding Asp401 was replaced by an alanine codon (GCC) in plasmid pVL1392-MBP-PGM [61]. To produce recombinant baculoviruses, the resulting plasmid (File S2) and those constructed previously to express MBP-Pgm, MBP-Pgm_ΔCR_ and MBP-Pgm_ΔCC_ [34] were each transfected into High Five insect cells together with BD BaculoGold Linearized Baculovirus DNA (BD Biosciences). The purification of MBP-tagged Pgm derivatives from recombinant baculovirus-infected cells was carried out as described [34], with a final elution step in buffer A supplemented with 10 mM maltose.

### NMR spectroscopy

High-quality NMR data were obtained for Pgm(692–768)*. NMR protein samples were concentrated to 0.15–0.5 mM in 90% H_2_O/10% D_2_O containing 5 mM HEPES, 25 mM NaCl, pH 6.8, or lyophilized and resuspended in 99.99% D_2_O. NMR experiments were performed at 293 K on 800 MHz or 950 MHz AVANCE III HD Bruker spectrometers equipped with TCI cryoprobes.

^1^H-^15^N HSQC spectra of Pgm(692–768)* were acquired at 800 MHz in 90% H_2_O/10% D_2_O with ^15^N carrier set to 120 ppm in order to observe the backbone amide groups. The INEPT half delay was set to 2.8 ms for observation of single-bond coupling (^1^J_HN_ ≈ 90 Hz).

Optimized long-range ^1^H-^15^N HSQC spectrum was recorded in 90% H_2_O/10% D_2_O with a 21 ms delay for the selective observation of the long-range proton (Hδ2 and Hε1)-nitrogen (Nδ1 and Nε2) correlations of histidines (^2^J_HN_ = 6–12 Hz) and the ^15^N carrier set to 200 ppm.

^1^H-^13^C HSQC spectra of Pgm(692–768)* were acquired in 99.9% D_2_O with ^13^C carrier set to 45 ppm and a half delay of 1.72 ms corresponding to a ^1^J_HC_ = 145 Hz, in order to observe the aliphatic protons. ^1^H-^13^C HSQC spectra with a half delay of 1.25 ms corresponding to a ^1^J_HC_ = 200 Hz were also recorded in order to observe the histidine Cδ2-Hδ2 and Cε1-Hε1 cross-peaks. In this case the ^13^C carrier was placed at 130 ppm with a spectral width of 40 ppm. A total of 256 data points were acquired with 4 transients per point with Echo-Antiecho quadrature in the indirect dimension.

Backbone (N, H, CO, Cα Cβ, Hα, Hβ) and side-chain (Cγ, Cδ, Cε, Hγ, Hδ, Hε) assignments were obtained using standard triple resonance assignment experiments [59] on a ^15^N-^13^C labeled protein sample: CBCA (CO) NH, HNCACB, HNCA, HN (CO) CA, HNCO and HN (CA) CO to assign backbone resonances, and H (CCO) NH, (H) C (CO) NH and (H)CCH-TOCSY to assign all the carbon and proton atoms in a given residue. Side-chain assignments were completed using a lyophilized sample redissolved into 99.99% D_2_O. ^15^N- and ^13^C-edited 3D NOESY experiments were acquired on samples in 90% H_2_O/10% D_2_O or 99.99% D_2_O with a mixing time of 150 ms to obtain nuclear Overhauser effect crosspeaks (NOEs) for structure determination. NMR data were collected and processed using TOPSPIN 3.5 software (Bruker) and analyzed using the CcpNmr software [41]. Backbone amide resonances were assigned for all non-proline amino acids except Lys740, for which no cross-peak was detected in the ^1^H-^15^N HSQC. We were able to assign 96 and 81% of the backbone and sidechain carbon atoms, respectively, 97 and 84% of the backbone and sidechain hydrogens, respectively, and 96% of the nitrogen atoms composing Pgm(692–768). Pgm(685–768)* assignments were deposited into BMRB (BMRB ID: **34527**).

Hydrogen–deuterium exchange experiments were acquired to determine residues protected from solvent through hydrogen bonding as follows. A 500-μL sample of 0.5 mM protein in the protonated NMR solution was lyophilized. The sample was resuspended in the equivalent volume of 99.99% D_2_O and quickly transferred to an NMR tube. A first ^1^H-^15^N HSQC was recorded in 20 min just after the dissolution, and a second one 3 days after the dissolution to observe hydrogen-to-deuterium exchange of backbone amide protons.

The ^15^N R_1_ and R_2_ relaxation rates and {^1^H}-^15^N heteronuclear NOE were measured at 20 °C on a 950 Avance III HD spectrometer equipped with a TCI cryoprobe. The ^15^N R1 and R2 relaxation experiments were based on the refocused ^1^H-^15^N HSQC relaxation experiments and recorded in an interleaved pseudo-3D method with an inter-scan delay of 5 s. For the determination of R_1_ relaxation rate constants, 13 total datasets were collected at relaxation delay times of 10, 50, 100, 200, 300, 400, 600, 800, 1100, 1500, 2000, 2500, 3000 ms. For the determination of R_2_ rate constants, 13 datasets were collected at delay times of 16.96, 33.92, 50.88, 67.84, 84.8, 101.76, 118.72, 135.68, 152.64, 169.60, 220.48, 271.36, 323.24 ms. R_1_ and R_2_ spectra were recorded as 128 × 2126 complex data points. For the backbone {^1^H}-^15^N heteronuclear NOE two different spectra were recorded as 512 × 2048 complex data points in an interleaved manner with and without a 5 s proton saturation pulse. The R_1_ and R_2_ rates and heteronuclear NOE values and their associated errors were determined from the peak intensities using the CcpNmr software [41]. Relaxation parameters were analyzed with the model-free formalism of Lipari and Szabo [42, 43], using the TENSOR2 program [62] to extract internal dynamical parameters: order parameter S^2^ describing the amplitude of the motions; internal correlation time τ_ε_ on the ps-ns timescale and R_ex_ reflecting exchange contribution on the μs–ms timescale. The isotropic tumbling model was selected since no improvement was found with the anisotropic model.

### NMR structure calculation

Inter-proton distance restraints were derived from the NOESY spectra (two-dimensional ^1^H NOESY and three-dimensional ^15^N- and ^13^C-NOESY). The Pgm(692–768)* structure was calculated in a semi-automated iterative manner using CYANA 2.1 [63]. Intra- and inter-residue NOEs were manually picked from the 3D NOESY experiments. The backbone dihedral angle restraints (_Φ_ and _Ψ_ angles) were generated using the chemical shift analysis software TALOS+ [40]. Hydrogen bond restraints were determined by hydrogen-deuterium exchange experiments and observation of NOE cross-peaks characteristic of α-helices and β-sheets. NOE peak lists, dihedral angle restraints, hydrogen bond restraints, and chemical shift assignments were used as input for CYANA 2.1. We used the standard CYANA protocol of seven iterative cycles of NOE assignment and structure calculation, followed by a final structure calculation. In each cycle, the structure calculation started from 200 randomized conformers, and the standard CYANA simulated annealing schedule was used with 10,000 torsion angle dynamics steps per conformer. The first calculations with only NOE restraints defined the general fold of the domain and revealed the two Zn^2+^ coordination modes. In the final refinement stage, distance restraints were added for Zn-Sγ (2.25–2.30 Å), Zn-Nδ1 (2.35–2.40 Å) and Zn-Nε2 (2.35–2.40 Å) and for the other bonds between the four height coordinating atoms, to ensure tetrahedral Zn^2+^ coordination geometry. Graphic representations were prepared with PyMOL [64]. The structures were deposited into the wwPDB (PDB ID **6ZOP**).

### In vitro DNA binding assays

Differential Radial Capillary Action of Ligand Assay (DRaCALA) is a simple and rapid filter binding assay allowing the detection of protein-ligand interactions [45]. This method includes no wash step, which represents an advantage over standard filter binding assays by avoiding sample loss and limiting complex dissociation. Briefly, a mixture of a protein and a labeled ligand is spotted directly onto a nitrocellulose membrane. When bound to the protein, the ligand remains at the center of the spot, while free unbound ligand moves by capillarity from the center to the periphery of the spot. We performed DRaCALA assays as described [19]. A ^32^P-labeled 80 bp double-strand substrate carrying IES 51A1835 from the surface antigen *A*^*51*^ gene was obtained following annealing of complementary top and bottom strand oligonucleotides (Eurofin MWG Genomics, Fig. S3). Only the top strand was labeled at its 5′ end using ^32^P-γ ATP and T4 polynucleotide kinase (New England Biolabs). Annealing was performed by heating to 95 °C followed by slow cooling to room temperature. The labeled dsDNA substrate (25 nM final concentration) was mixed in 25 mM HEPES pH 7.5, 0.1 mg/ml BSA, 0.5 mM DTT and 100 mM NaCl-containing buffer with an excess of each purified protein at the following final concentrations: GST (5 μM); N-terminal GST fusions for PB(538–594), Pgm(692–768), PgmL2(540–614) and PgmL4a(856–931, 1 to 3 μM), N-terminal MBP fusions for PgmL1(463–539), PgmL3a(471–550) or MBP-PgmL5a(761–840, 400 to 900 nM); full-length Pgm or its mutant derivatives (400 nM). Complexes were loaded onto a Nitrocellulose Hybond ECL membrane. Following air drying, the membrane was exposed to a Phosphorimager screen, which was scanned using a Typhoon scanner (GE Healthcare Life Sciences).

Electrophoretic Mobility Shift Assays (EMSA) using purified CRDs were performed as described [35], using the same ^32^P-labeled 80-bp dsDNA substrate as above for IES 51A1835 and a ^32^P-labeled 70-bp dsDNA fragment carrying the left end of IES 51A4404 and its flanking MAC-destined sequences (Fig. S3). After electrophoresis in a 5% acrylamide, 0.5x TBE gel, free DNA and protein-DNA complexes were visualized following exposure of the dried gel to a Phosphorimager screen (see above).

### NMR analyses of the interaction of Pgm(692–768)* with DNA and histone H3

Two double-stranded DNA substrates (Eurofins Genomics, Ebersberg, Germany) (Fig. S3), were used in NMR interaction studies. They were obtained through annealing of complementary oligonucleotides, by heating to 95 °C followed by slow cooling to room temperature. Proper annealing was confirmed by the presence of imino protons at 12 to 14 ppm in 1D ^1^H-NMR spectra (Fig. S12). The interactions between these DNA molecules with Pgm(692–768)* were probed by ^1^H-^1^H NOESY experiments with excitation sculpting water suppression [65] with 200 ms mixing time on an AVANCE Bruker 950 MHz spectrometer, with a spectral width of 18,043 Hz with 2202 complex points in t2 and 688 t1-increments. Spectra were acquired on Pgm(692–768)* in the absence and presence of an equimolar amount of DNA.

A titration of 100 μM ^15^N-Pgm(692–768)* with 50 to 1000 μM of the H3(1–19) histone peptide was followed by ^1^H-^15^N-HSQC in 5 mM HEPES pH 6.8, 25 mM NaCl, 95% H_2_O / 5% D_2_O at 20 °C, acquired on an AVANCE Bruker 800 MHz spectrometer with 24 scans, 11,160 Hz and 2 k complex points, and 2513 Hz and 240 points in the ^1^H and ^15^N dimensions, respectively. A π/2 phase-shifted squared sine bell window function was applied before the FT. The dissociation constant (K_D_) was determined by least squares fitting of chemical shift changes between free and bound states (Δδ_obs_) to the non-linear equation (adapted from [66]):

$$ {\Delta \delta}_{obs}={\Delta \delta}_{max}\frac{\left({K}_D+{\left[H3\right]}_0+{\left[P\right]}_0\right)-\sqrt{{\left({K}_D+{\left[H3\right]}_0+{\left[P\right]}_0\right)}^2-4\left({\left[P\right]}_0{\left[H3\right]}_0\right)}}{2{\left[P\right]}_0} $$

where Δδ_obs_ and Δδ_max_ are the measured and maximum chemical shift perturbations for a given resonance, respectively; and [H3]_0_ and [P]_0_ are the H3(1–19) and Pgm(692–768)* concentrations, respectively.

The final titration point (100 μM Pgm(692–768)* and 1000 μM H3(1–19)) was used to observe the effect of this interaction on the peptide. ^1^H-^1^H TOCSY experiments were acquired on this sample and a 1000 μM sample of H3(1–19) in the same buffer at pH 6.8. These experiments were acquired on a Bruker 800 MHz spectrometer with 60 ms mixing time, 11,160 Hz spectral width, 96 scans, 2 k and 512 complex points in the ^1^H and ^15^N dimensions, respectively.

### *Paramecium* cell culture, nuclei isolation and histone extraction

Autogamous cultures of *Paramecium tetraurelia* strain 51new [67] were grown according to [68]. Ten hours after 50% of cells became autogamous, they were harvested by centrifugation. The resulting cell pellet was flash-frozen in liquid nitrogen and stored at − 80 °C prior to nuclear extraction. The cell pellet was thawed on ice with 2 volumes of lysis buffer (0.25 M sucrose; 10 mM MgCl_2_; 10 mM Tris pH 6.8; 0.2% Nonidet P40; 4X CalbioChem protease inhibitor cocktail set I). Cells were lysed with a Potter-Elvehjem homogenizer. The lysate was placed in an Eppendorf tube and subsequently centrifuged at 1000 g for 2 min in a swinging centrifuge and the nuclei-containing pellet was washed 3 times in 5 volumes of wash buffer (0.25 M sucrose, 10 mM MgCl_2_; 10 mM Tris pH 6.8; 4X CalbioChem protease inhibitor cocktail set I). Acid extraction of histones from the nuclei was performed according to [69]. The purity of histone preparations was monitored on SDS gels (Fig. S4) and their protein concentration was measured using a BCA assay. Around 100 μg of histones were extracted from a 1-L cell culture.

### Glutathione S-transferase (GST) and maltose binding protein (MBP) pull-down assays

Expression of GST-CRD and MBP-CRD fusions was carried out as described above. Four hundred μl of MBP/MBP-CRD-containing lysates or 200 μl of 1/10 diluted GST/GST-CRD-containing lysates in buffer B were loaded onto 100 μL pre-equilibrated slurry glutathione sepharose beads 4B (GE healthcare) or amylose resin (New England Biolabs), respectively, and incubated for 1 h at 4 °C. Beads were then washed 3 times in 200 μl of buffer B. Five μg of *Paramecium* histones were diluted in 100 μl of buffer B, added to the beads and incubated overnight on a rotating wheel at 4 °C. Because buffer B contains 500 mM NaCl, we can exclude that any observed histone/CRD interaction is indirectly mediated through DNA binding, because, as shown by NMR and DRaCALA experiments (Fig. 5), no interaction is detected between the Pgm CRD and DNA in the presence of as little as 100 mM NaCl. After incubation, the beads were washed 4 times with 200 μl of buffer B (last wash on wheel for 10 min at 4 °C). The beads (~ 50 μL) were boiled for 10 min following addition of 25 μl 4X Laemmli sample buffer (Bio-Rad) supplemented with 1.4 M β-mercaptoethanol.

### Enzyme-linked immunosorbent assays (ELISA)

Each well of a Pierce streptavidin coated high capacity 96-well plate was washed 3 times with 200 μL of buffer C (25 mM Tris pH 7.4; 150 mM NaCl; 0.1% BSA; 0.05% Tween 20). C-terminally biotinylated H3 peptides (4 μM in 100 μL Wash Buffer) were applied into wells to saturate their surface and the plate was incubated at room temperature for 1 h. The plate was washed three times with 200 μL buffer C and then 100 μL of purified MBP-CRD or GST-CRD fusion proteins (10 to ~100 nM) were added to each well. The plate was incubated at 4 °C overnight, then washed as above to remove unbound proteins. One hundred μL of monoclonal anti-MBP-HRP antibody (NEB #E8038S; diluted 1/10,000 in buffer C) was added to each well and the plate was incubated for 30 min at room temperature and then washed three times. Peroxidase enzyme activity was measured with tetramethylbenzidine (1-Step™ Ultra TMB-ELISA, ThermoScientific) using a TECAN Infinite 200 pro reader at 450 nm.

### Microwave plasma atomic emission spectroscopy

The relative amount of zinc complexed to GST-fused Pgm(692–768) and its mutant versions carrying the H701S, H738S or C712S mutations was determined using microwave plasma atomic emission spectroscopy. Proteins were extracted from the bacterial lysate using glutathione-sepharose beads and washed with lysis buffer as described above. The beads were then washed with 2 mM EDTA to remove excess non-complexed zinc. They were then treated with nitric acid to dissolve the glutathione-sepharose beads. The solutions were analyzed by microwave plasma atomic emission spectroscopy using an Agilent 4200 MP-AES spectrometer. Zinc concentrations were determined by comparison to a standard curve and normalized with respect to initial protein concentration.

## Supplementary Information

**Additional file 1: Figure S1**. Impact of EDTA on the structuration of Pgm(692–768). 1D ^1^H spectra recorded on 250 μM of Pgm(692–768)* at 800 MHz in 5 mM Hepes pH 6.8, 25 mM NaCl at 293 K. (a) Amide and aromatic region of the 1D ^1^H proton and (b) aliphatic region of the 1D ^1^H proton, in the absence of EDTA (blue: a1 and b1) and in the presence of 2 mM (red: a2 and b2) or 10 mM EDTA (green: a3 and b3). **Figure S2**. Superposition of 15 structures generated using CYANA. Superposition on the (701–751) backbone atoms. The flexible N- and C-terminal arms are in light blue and pink, respectively. The two β-sheets are colored in magenta and blue and the two longest α-helices in orange. Zn^2+^ ions are represented by purple spheres. **Figure S3**. Double-strand DNA substrates used for in vitro DNA binding assays. Red: IES sequence. Black: Flanking MAC-destined sequences. The conserved TA dinucleotides at IES boundaries are in bold. **Figure S4**. Control experiments for the *Paramecium* histone pull-down assays. (a) Acid extraction of endogenous histones from *P. tetraurelia*. Non-soluble and soluble fractions of *Paramecium* histones were revealed on a western blot by Ponceau staining (left) and immunodetection with α-Histone H3 antibodies (right) after migration on a 5–15% polyacrylamide Tris-Glycine SDS gel and transfer. (b) Non-soluble and soluble fractions of histones were revealed with InstantBlue (Sigma) after migration on a 15% polyacrylamide Tris-Glycine SDS gel (left) and on a western blot by immunodetection with α-histone H3 antibodies (right). (c) Pulldown assays with empty beads, beads bound by GST alone or the wild type GST-Pgm CRD fusion and acid extracted histones shown in panel a. (d) Pulldown assays with MBP alone or the wild type MBP-Pgm CRD fusion and acid extracted histones shown in panel b. When indicated, Triton was added to buffer B to reach a final concentration of 2%. Under these conditions, no H3 was found in the precipitate. (e) Pulldown assays of GST, GST-CRD wild type, GST-CRD(C712S) single mutant or GST-CRD(H701S + C712S) double mutant. **Figure S5**. Interaction of the PgmL CRDs with H3(1–19) and H3(16–35). For H3(1–19), ELISA assays were performed using unmodified H3(1–19)-b, trimethylated H3(1–19)K4me3-b and H3(1–19)K9m3-b and the Scrambled 2 control peptide. For H3(16–35), ELISA assays were performed using unmodified H3(16–35)-b and trimethylated H3(16–35)K27me3-b. Histone peptides were incubated with 20 nM MBP alone or with 10 to 20 nM MBP-PgmL CRDs. Error bars represent the standard deviation between technical triplicates. **Figure S6**. Interaction of wild-type and mutant Pgm CRDs with histone H3. (a) In vitro pulldown using GST alone, wild-type GST-Pgm(692–762) (CRD WT), single mutant GST-Pgm(692–762) carrying the C712S substitution (CRD C712S) and the double mutant GST-Pgm(692–762) carrying the H701S and C712S substitutions (CRD H701S + C712S), with acid extracted histones from *P. tetraurelia* (for the control of acid extracted histone preparation and full size blots see **Figure S4**). (b) Ratio of the quantified volume of bound versus input H3 bands from the in vitro pulldown assay. (c) ELISA assays using 100, 20 and 10 nM of GST alone or GST fused to CRD WT, CRD C712S and CRD H701S + C712S with the H3(1–19)-b peptide. (d) ELISA assays using ~ 100 nM of MBP alone or MBP fused to CRD WT or CRD H701S + C712S with H3(1–19)-b. MBP fusion proteins where loaded into the wells either in absence of EDTA (left) or in the presence of 10 mM EDTA (right). Error bars represent the standard deviation between technical triplicates. For the control of MBP and GST fusions used in ELISA see **Figure S11**. **Figure S7**. Evidence for an interaction between the Pgm CRD and the N terminus of H3(1–19). (a) ELISA assays with 20 nM MBP or MBP-Pgm(692–768) (MBP-CRD) against unmodified H3(1–19)-b, trimethylated H3(1–19)K4me3-b and H3(1–19)K9m3-b, control peptides Scrambled 1 (*: TECAN absorbance detection at 450 nm close to saturation) and Scrambled 2, unmodified H3(16–35)-b and trimethylated H3(16–35)K27me3-b. Error bars represent the standard deviation between technical triplicates. (b) Selected region of the ^1^H-^1^H TOCSY spectrum of 1 mM H3(1–19) in the absence (in black) and presence of 0.1 mM Pgm(692–768)* (red) at pH 6.8, 20 °C and 800 MHz ^1^H. The resonances of N-terminal residues Ala1 and Arg2 are shifted by this interaction (black labels) while the resonances of other residues do not appear to be shifted (grey labels). **Figure S8**. Comparison of the folding of different CRDs. (a) The PHD domain of human BPTF in complex with H3(1–15)K4me3 peptide (PDB ID 3FUU) provides an example of the interaction between a PHD domain and a histone tail. The H3(1–7) N-terminal domain of histone H3 is colored in green. (b) The Pgm CRD, in which the regions contacting the H3 tail are highlighted in blue. (c) The PB CRD. In all panels, the β-sheets that may be accessible for an interaction with histone tails are colored in orange. Zn^2+^ ions are represented by purple spheres. **Figure S9**. Comparison of the structure of typical and atypical C1 domains. (a) Typical C1 domain of protein kinase Cδ complexed with phorbol ester (PDB id 1PTR). The phorbol ester is drawn as pink sticks. (b) Atypical C1 domain of the Rho-associated protein kinase 2 ROCK II (PDB id 2ROW). Typical and atypical C1 domains adopt similar folds but differ in their number of β strands. In contrast to the typical C1 domain, which has a single β sheet, the atypical C1 domain of ROCK II harbors two β sheets and is therefore more similar to the Pgm CRD. **Figure S10**. Comparison of the long bent hairpin present in Pgm and in typical and atypical C1 domains. The Pgm(706–720) hairpin is displayed in a1 and a2, the typical C1 domain of the cys2 activator-binding domain of protein kinase C delta (PDB ID:1PTR) in b1 and b2 and the atypical C1 domain of the Rho kinase II (PDB ID 2ROW) in c1 and c2. Acidic residues are colored in red, basic residues in blue, hydrophobic residues in grey and all other residues are in green. **Figure S11**. Expression and purification of the wild-type and mutant CRDs used in this study. (a) 15% SDS-PAGE gel summarizing the purification steps used to produce the wildtype Pgm(692–768)* peptide for NMR experiments. 1: molecular weight marker; 2: total cell extract before induction with 0.2 mM IPTG; 3: total cell extract after IPTG induction; 4: clear supernatant; 5: glutathione Sepharose beads, unbound fraction; 6: glutathione Sepharose beads, bound fraction; 7: PreScission cleavage supernatant; 8–14: elution fractions from Hitrap Q HP. (b) Expression and purification of wild-type and mutant GST-Pgm(692–768) for DNA and histone binding assays. (c) Purified preparations of MBP-PgmL1(463–539), MBP-PgmL2(540–614), MBP-PgmL3a(471–550), MBP-PgmL4a(856–931), MBP-PgmL5a(761–840), MBP-Pgm(692–768) and MBP used for DNA and histone binding assays. (d) Purified preparations of MBP alone, MBP-Pgm(692–768) (MBP-CRD WT) and MBP-Pgm(692–768) double mutant (MBP-CRD H701S + C712S) used for ELISA assays. For those preparations, in which some of the free MBP tag was released upon over-expression or during bacterial lysis, only the full-length fusion protein was taken into account to calculate the amount of input CRD in histone-binding assays. (e) Effect of alkylation agent N-ethyl-maleimide (NEM) on intramolecular S-S bond formation in the wildtype Pgm CRD. MBP, MBP-CRD WT or MBP-CRD H701S + C712S were preincubated for 10 min with (+) or without (−) 20 μM NEM before denaturation (boiling in 1X Laemmli buffer + 357 mM β-mercaptoethanol) and loading on an SDS-PAGE gel. NEM-induced disappearance of the lower band of the MBP-CRD WT doublet indicates that the doublet (observed in panels b, d and e) results from intramolecular disulfide bond formation within the wildtype Pgm CRD. Panels b to e show 5–15% SDS-PAGE gels. **Figure S12**. ^1^H-NMR spectra of imino protons of i1835 (blue) and i4404 (red). Their presence shows that DNA was correctly annealed prior to interaction studies. i1835: CTACTACATAATGCTAAACTCATTTATAGATGGATTGTTTTCCAAGTATCTATATC and its complementary strand; i4404: GCTTGCACATCTCTAGTTGATGG and its complementary strand (see **Figure S4**b). **Table S1**. Comparison of zinc-binding capability of histidine mutants of Pgm(692–768). Microwave-plasma atomic emission spectroscopy was used to determine the amount of zinc in wild-type (wt) and histidine-to-serine mutants of GST-fused Pgm(692–768). Zinc concentrations were determined both with and without a washing step with 2 mM EDTA.

## Data Availability

Pgm(685–768)* assignments were deposited into BMRB (BMBR ID: **34527**). The structures were deposited into the wwPDB (PDB ID **6ZOP**). All the datasets used and analyzed during the current study are available from the corresponding authors on reasonable request.

## Abbreviations

TEs: Transposable elements
TIRs: Terminal inverted repeats
*T. ni*: *Trichoplusia ni*
PB: PiggyBac transposase from *T. ni*
PBLE: *PiggyBac*-like elements
PGBD: *PiggyBac* transposable element-derived
MIC: Micronucleus
MAC: Macronucleus
IESs: Internal eliminated sequences
Pgm: PiggyMac
PgmL: PiggyMac-like
CRD: Cysteine-rich domain
NMR: Nuclear magnetic resonance
