# Supplementary material for: The unusual structure of the PiggyMac cysteine-rich domain reveals zinc finger diversity in PiggyBac-related transposases
Source: Mob DNA. 2021 Apr 29;12:12. doi: 10.1186/s13100-021-00240-4 (PMC8086355; doi:10.1186/s13100-021-00240-4)
Supplement: Supplementary file 1 — Additional file 1: Figure S1. Impact of EDTA on the structuration of Pgm(692–768). 1D 1H spectra recorded on 250 μM of Pgm(692–768)* at 800 MHz in 5 mM Hepes pH 6.8, 25 mM NaCl at 293 K. (a) Amide and aromatic region of the 1D 1H proton and (b) aliphatic region of the 1D 1H proton, in the absence of EDTA (blue: a1 and b1) and in the presence of 2 mM (red: a2 and b2) or 10 mM EDTA (green: a3 and b3). Figure S2. Superposition of 15 structures generated using CYANA. Superposition on the (701–751) backbone atoms. The flexible N- and C-terminal arms are in light blue and pink, respectively. The two β-sheets are colored in magenta and blue and the two longest α-helices in orange. Zn2+ ions are represented by purple spheres. Figure S3. Double-strand DNA substrates used for in vitro DNA binding assays. Red: IES sequence. Black: Flanking MAC-destined sequences. The conserved TA dinucleotides at IES boundaries are in bold. Figure S4. Control experiments for the Paramecium histone pull-down assays. (a) Acid extraction of endogenous histones from P. tetraurelia. Non-soluble and soluble fractions of Paramecium histones were revealed on a western blot by Ponceau staining (left) and immunodetection with α-Histone H3 antibodies (right) after migration on a 5–15% polyacrylamide Tris-Glycine SDS gel and transfer. (b) Non-soluble and soluble fractions of histones were revealed with InstantBlue (Sigma) after migration on a 15% polyacrylamide Tris-Glycine SDS gel (left) and on a western blot by immunodetection with α-histone H3 antibodies (right). (c) Pulldown assays with empty beads, beads bound by GST alone or the wild type GST-Pgm CRD fusion and acid extracted histones shown in panel a. (d) Pulldown assays with MBP alone or the wild type MBP-Pgm CRD fusion and acid extracted histones shown in panel b. When indicated, Triton was added to buffer B to reach a final concentration of 2%. Under these conditions, no H3 was found in the precipitate. (e) Pulldown assays of GST, GST-CRD wild t [file 13100_2021_240_MOESM1_ESM.zip › File S1 fasta CRDs for Figure1.docx]

>PiggyBat(512-574)_Mlu_Mitra PNAS 2013_<https://doi.org/10.1073/pnas.1217548110>

DMRKHTLQAIVGSGKKKNILRRCRVCSVHKLRSETRYMCKFCNIPLHKGACFEKYHTLKNYLE

>PB-Tni(547-594)_AAA87375

STEEPVMKKRTYCTYCPSKIRRKANASCKKCKKVICREHNIDMCQSCF

>Cag(550-599)_ADV17598

DTSFDEPEPKKRRYCGFCSYKKKRMTKTQCFKCKKPVCGEHNIDVCQDCI

>Har(550-597)_ABS18391

ISNEPEPKKRRYCGVCSYKKRRMTKAQCCKCKKAICGEHNIDVCQDCI

>Tru_Pigibaku1(574-624)_XP_011603527.1

AEPEVVNTSNKKKRCEVCGPKMDRKTQYTCIKCKKYICNTHTVKLCPSCVV

>Oni(510-557)_XP_005458919_PGBD4 ?

PSPIIKCKGRRQCELCKEKRRRIVNTCCKCEKYTCKDHSVSICNNCSA

>Pny(512-561)_XP_005755147_PGBD4 ?

AGPSGLTHPKGRKRCELCCDYMRRVGNSCSKCGRFTCRAHSKFICSHCST

>Bmo(555-610)_BAD11135

PSPRHVNVPGRYVRCQDCPYKKDRKTKRSCNACAKPICMEHAKFLCENCAELDSSL

>Ago(518-577)_ADU04477

SDVSTTPPSKRGTCFECGRKKNAATSMKCTKCMRFVCKLHSKKIIICEKCSNNDDNGNSE

>Hvi(524-581)_ABD76335

NAEVQDPGSTSRGGPSTSYKRCHICPRSKDKKIRFMCAKCHHHICHDHSTMICDKCID

>PLE-wu(654-708)_ref ?

RFSNVGDHMPNDIPSYQRCRYCSTKAKDKRSKIKCSKCGVPLCITPCFSNFHKQV

>Pgbd2-Hs(545-592)_NP_733843.1

SRFDMIGHWIIHQDKRTRCALCHSQTNTRCEKCQKGVHAKCFREYHIR

>Pgbd3-Hs(546-593)_NP_736609.2

SRYDGINHVIVKQGKQTRCAECHKNTTFRCEKCDVALHVKCSVEYHTE

>Pgbd4-Hs(517-585)_NP_689808.2

LRLSGRHFPKSIPATSGKQNPTGRCKICCSQYDKDGKKIRKETRYFCAECDVPLCVVPCFEIYHTKKNY

>Tpb2(599-663)_TTHERM_01107220

KNCDYSAHILVRSRTKKKSCIECKQLTLFSCSTCSNMFKMRIPLCQSGFNQCYDFHASKTYEEVV

>Pgm(692-768)_PTET.51.1.P0490162

FSYFAKIQPHTFIEGEEIVKCSECGNETKVFCQECTILKAEVVGLCHEKDTIKCQRFHEFMDFELDKNKEVIDKRKG

>Tpb7(717-773)_TTHERM_00616500

HFWEENQGGKKQECIVCHTKTRNYCIQCSEKKKQIIGFCGNSNCLQKHNELPAKLLN

>PgmL4a(864-924)_PTET.51.1.P0340197

QFPQNHTLESGDTGTFSCIECGESSQTICRECSNHFQMLIPVCRSKNEQCLRSHIEMLASQ

>PgmL4b(862-922)_PTET.51.1.P0480099

QFPQNHTLESGDTGTFSCIECGESSQTICRECSNHFQMLIPVCRSKNEQCLKSHIEMLVNQ

>PgmL5a(767-829)_PTET.51.1.P0570051

DQLYHCPIHNGNARCQVCLSKSILSKTTASCLGCNKVLGTNIFLCIYPCFRLFHLNPKLYLKE

>PgmL5b(764-826)_PTET.51.1.P0510172

DQLYHCPIHNGNARCQVCLSKSILSKTTASCLGCNKVLGTNIFLCIYPCFRLFHLNPKLYLKE

>Lia5(764-830)_TTHERM_00653910

NIRQGGTHVQKKDGKQGICLVCLQEKNIQNNTFITCQECSLQNKKPVYLCDKCFEVYHLEINVNRDN

>Tpb1(1146-1199)_TTHERM_000309879

HFLERQNQVGLCSLCKQATFFTCESCNYGNKKIALCPVNCHKEHMKKVYNLID

>Tpb6(1473-1531)_DAA80465.1_MICspecific

FQLNHFVIFSKYKQKCCICKKITKFACDTCIDPLLNQKLNLCPGFCQKTHMLSFFKNQK

>PgmL2(541-611)_PTET.51.1.P0380073

ELLPKINFHVPRKCNPPAKLQNKLVCLVCKKVPTEMVECESCSEISGKLITLCATECFSLFHQEPKKYVQS

>PgmL1(458-528)_PTET.51.1.P0110267

KINTKSLFHIPTIMATPKTYAGSMNCLVCKRNTQLITLCKPCSEISGKLVILCACDCFYLFHQNTLEYIIN

>PgmL3c(474-541)_PTET.51.1.P0020217

SESYHTPIPQKQEESFKYCVVCMKFNQLTKPFYRCRLCEKLLNENKIFICAFPCFELFHRNPSQFIEC

>PgmL3b(473-540)_PTET.51.1.P0080308

ADQFHTPIPQKQEDTFKYCLVCMKFEGLTKPYYRCQLCEKILNVNKIFLCPFPCFELFHKNPSDFVDC

>PgmL3a(472-539)_PTET.51.1.P0010374

ADQFHTPIPQKQEDTFKYCLVCMKFEGLTKPYYRCQLCEKLLKVNKIFLCPFPCFELFHRNPSDFMVC
