# Supplementary material for: The unusual structure of the PiggyMac cysteine-rich domain reveals zinc finger diversity in PiggyBac-related transposases
Source: Mob DNA. 2021 Apr 29;12:12. doi: 10.1186/s13100-021-00240-4 (PMC8086355; doi:10.1186/s13100-021-00240-4)
Supplement: Supplementary file 1 — Additional file 1: Figure S1. Impact of EDTA on the structuration of Pgm(692–768). 1D 1H spectra recorded on 250 μM of Pgm(692–768)* at 800 MHz in 5 mM Hepes pH 6.8, 25 mM NaCl at 293 K. (a) Amide and aromatic region of the 1D 1H proton and (b) aliphatic region of the 1D 1H proton, in the absence of EDTA (blue: a1 and b1) and in the presence of 2 mM (red: a2 and b2) or 10 mM EDTA (green: a3 and b3). Figure S2. Superposition of 15 structures generated using CYANA. Superposition on the (701–751) backbone atoms. The flexible N- and C-terminal arms are in light blue and pink, respectively. The two β-sheets are colored in magenta and blue and the two longest α-helices in orange. Zn2+ ions are represented by purple spheres. Figure S3. Double-strand DNA substrates used for in vitro DNA binding assays. Red: IES sequence. Black: Flanking MAC-destined sequences. The conserved TA dinucleotides at IES boundaries are in bold. Figure S4. Control experiments for the Paramecium histone pull-down assays. (a) Acid extraction of endogenous histones from P. tetraurelia. Non-soluble and soluble fractions of Paramecium histones were revealed on a western blot by Ponceau staining (left) and immunodetection with α-Histone H3 antibodies (right) after migration on a 5–15% polyacrylamide Tris-Glycine SDS gel and transfer. (b) Non-soluble and soluble fractions of histones were revealed with InstantBlue (Sigma) after migration on a 15% polyacrylamide Tris-Glycine SDS gel (left) and on a western blot by immunodetection with α-histone H3 antibodies (right). (c) Pulldown assays with empty beads, beads bound by GST alone or the wild type GST-Pgm CRD fusion and acid extracted histones shown in panel a. (d) Pulldown assays with MBP alone or the wild type MBP-Pgm CRD fusion and acid extracted histones shown in panel b. When indicated, Triton was added to buffer B to reach a final concentration of 2%. Under these conditions, no H3 was found in the precipitate. (e) Pulldown assays of GST, GST-CRD wild t [file 13100_2021_240_MOESM1_ESM.zip › 2021-03-16_Sup Materials final Mobile DNA.pdf]

SUPPLEMENTAL INFORMATION

The unusual structure of the PiggyMac cysteine-rich domain reveals zinc finger diversity in PiggyBac-related transposases

Marc Guérineau*, Luiza Bessa*, Séverine Moriau*, Ewen Lescop, François Bontems, Nathalie Mathy, Eric Guittet, Julien Bischerour, Mireille Bétermier#, Nelly Morellet#

SUPPLEMENTARY FIGURES

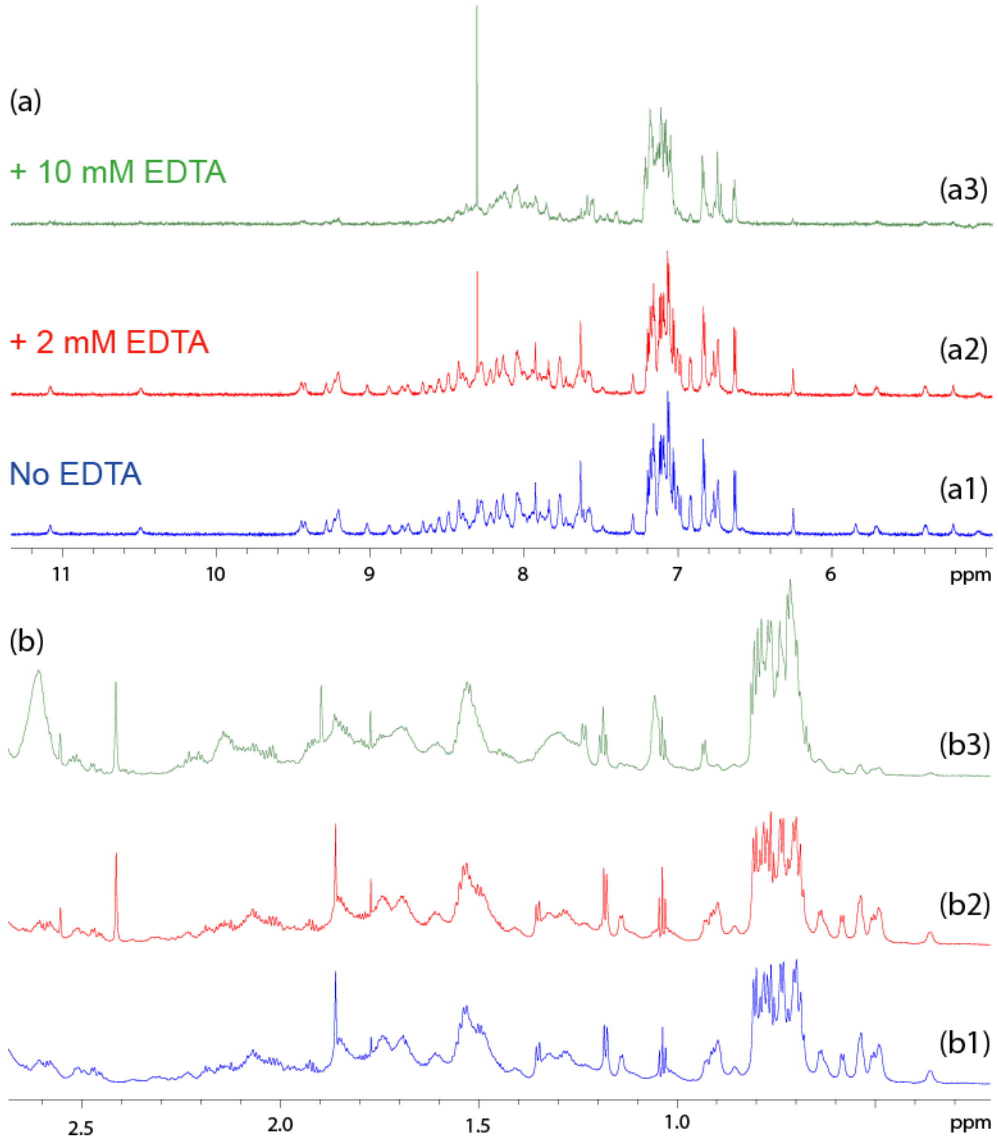

Fig. S1. **Impact of EDTA on the structuration of Pgm(692-768).** 1D ^{1}H spectra recorded on 250 µM of Pgm(692-768)* at 800 MHz in 5 mM Hepes pH 6.8, 25 mM NaCl at 293 K. (a) Amide and aromatic region of the 1D ^{1}H proton and (b) aliphatic region of the 1D ^{1}H proton, in the absence of EDTA (blue: a1 and b1) and in the presence of 2 mM (red: a2 and b2) or 10 mM EDTA (green: a3 and b3).

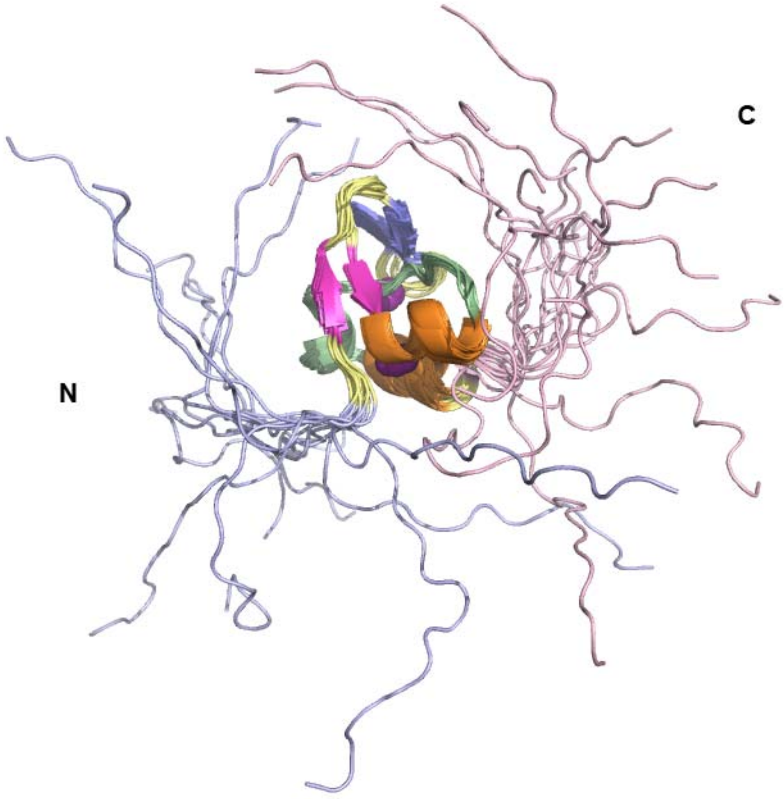

Fig. S2. Superposition of 15 structures generated using CYANA. Superposition on the (701-751) backbone atoms. The flexible N- and C-terminal arms are in light blue and pink, respectively. The two β-sheets are colored in magenta and blue and the two longest α-helices in orange. Zn^{2+} ions are represented by purple spheres.

(a) DRaCALA and EMSA

IES *51A*1835

5′-ACTACTACATAATGCTAAACTCATT**TA**TAGATGGATTGTTTTCCAAGTATCTA**TA**TCATCATGTGTTGCTAATAACCCTG-3′ (Top)
3′-TGATGTATTACGATTTGAGTAA**AT**ATCTACCTAACAAAAGGTTCATAGAT**AT**AGTAGTACACAACGATTATTGGGACGCTA-5′ (Bottom)

IES *51A*4404 (left end and flanking DNA)

5′-ctAGCTTACAACGCAGCTTGCACATCTCTAGTTGATGG**TA**TTGTGGTGTTATTTTAAAAAGTAATATATT-3′ (Top)
3′-GAATGTTGCGTCGAACGTGTAGAGATCAACTACC**AT**AACACCACAATAAAATTTTTCATTATATAAtagc-5′ (Bottom)

(b) NMR

IES *51A*1835 (i1835)

5′-CTACTACATAATGCTAAACTCATT**TA**TAGATGGATTGTTTTCCAAGTATCTA**TA**TC-3′
3′-GATGTATTACGATTTGAGTAA**AT**ATCTACCTAACAAAAGGTTCATAGAT**AT**AGTAG-5′

IES *51A*4404 (i4404, left flanking DNA)

5′-GCTTGCACATCTCTAGTTGATGG-3′
3′-CGAACGTGTAGAGATCAACTACC-5′

Fig. S3. Double-strand DNA substrates used for *in vitro* DNA binding assays. Red: IES sequence. Black: Flanking MAC-destined sequences. The conserved TA dinucleotides at IES boundaries are in bold.

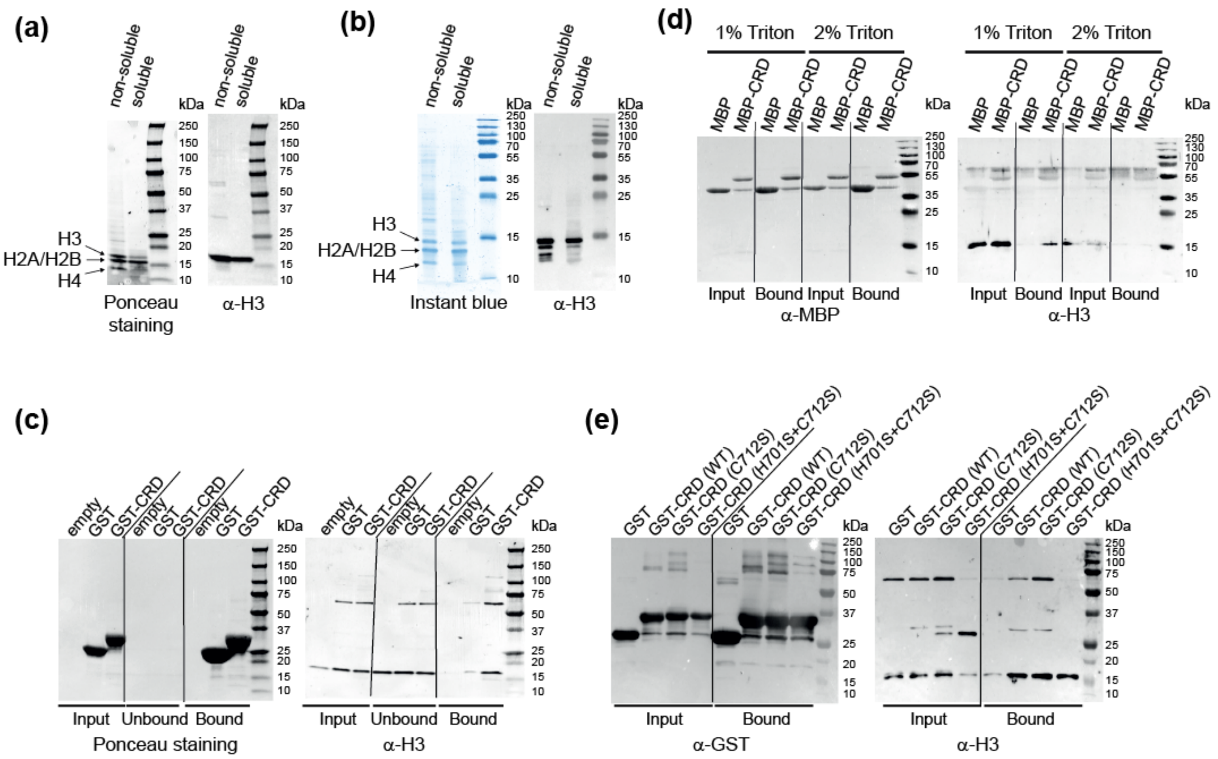

Fig. S4. Control experiments for the *Paramecium* histone pull-down assays. (a) Acid extraction of endogenous histones from *P. tetraurelia*. Non-soluble and soluble fractions of *Paramecium* histones were revealed on a western blot by Ponceau staining (left) and immunodetection with α-Histone H3 antibodies (right) after migration on a 5-15% polyacrylamide Tris-Glycine SDS gel and transfer. (b) Non-soluble and soluble fractions of histones were revealed with InstantBlue (Sigma) after migration on a 15% polyacrylamide Tris-Glycine SDS gel (left) and on a western blot by immunodetection with α-histone H3 antibodies (right). (c) Pulldown assays with empty beads, beads bound by GST alone or the wild type GST-Pgm CRD fusion and acid extracted histones shown in panel a. (d) Pulldown assays with MBP alone or the wild type MBP-Pgm CRD fusion and acid extracted histones shown in panel b. When indicated, Triton was added to buffer B to reach a final concentration of 2%. Under these conditions, no H3 was found in the precipitate. (e) Pulldown assays of GST, GST-CRD wild type, GST-CRD(C712S) single mutant or GST-CRD(H701S+C712S) double mutant.

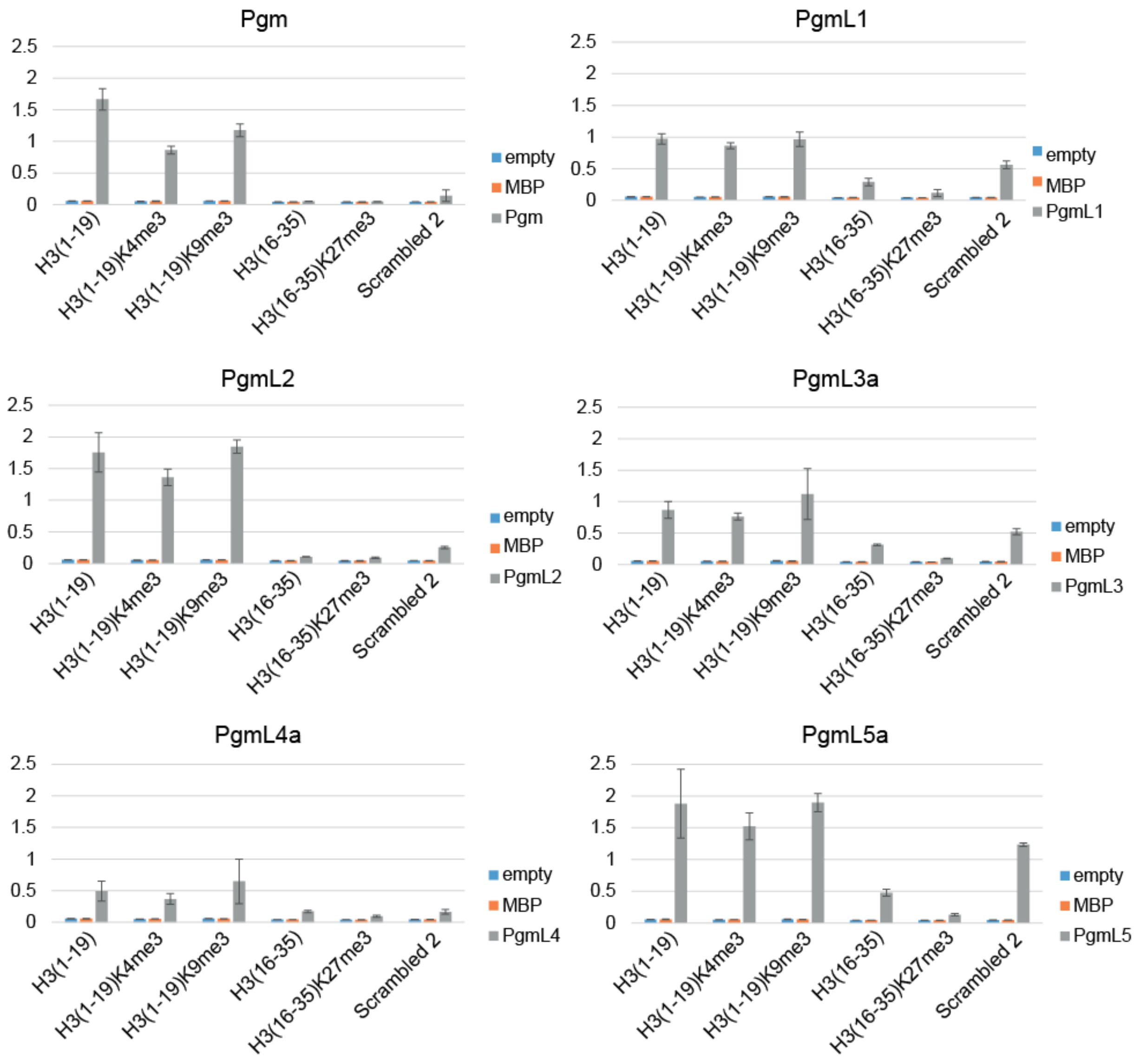

Fig. S5. Interaction of the PgmL CRDs with H3(1-19) and H3(16-35). For H3(1-19), ELISA assays were performed using unmodified H3(1-19)-b, trimethylated H3(1-19)K4me3-b and H3(1-19)K9m3-b and the Scrambled 2 control peptide. For H3(16-35), ELISA assays were performed using unmodified H3(16-35)-b and trimethylated H3(16-35)K27me3-b. Histone peptides were incubated with 20 nM MBP alone or with 10 to 20 nM MBP-PgmL CRDs. Error bars represent the standard deviation between technical triplicates.

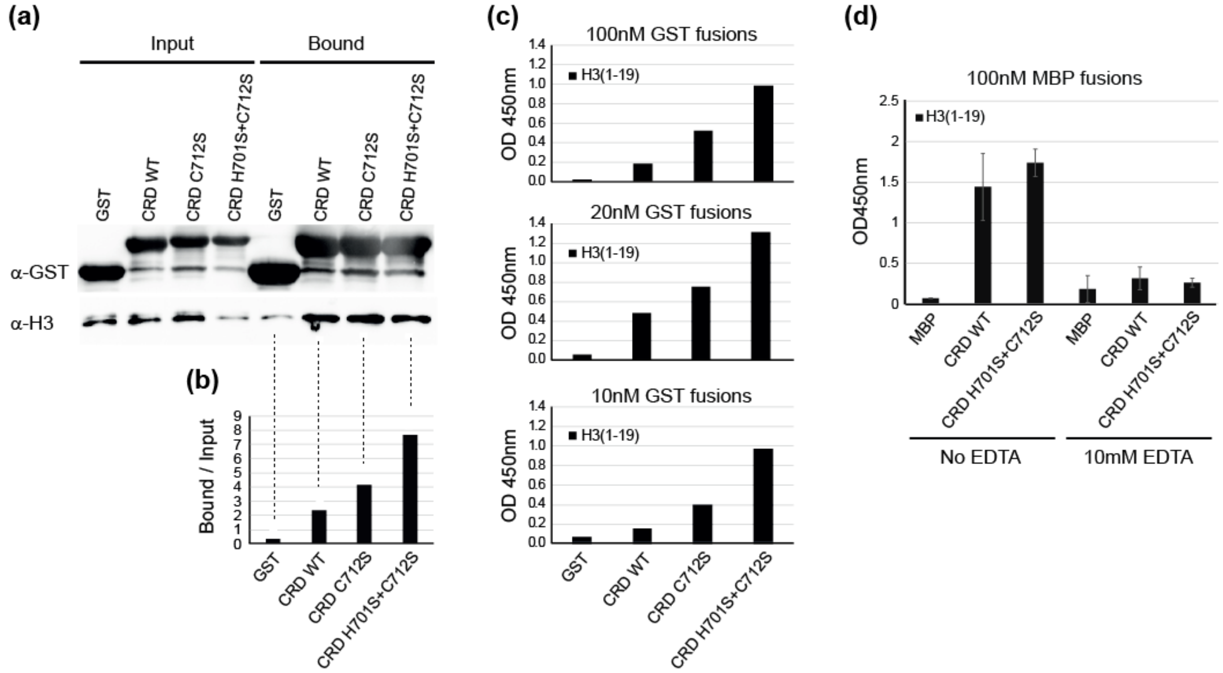

Fig. S6. Interaction of wild-type and mutant Pgm CRDs with histone H3. (a) *In vitro* pulldown using GST alone, wild-type GST-Pgm(692-762) (CRD WT), single mutant GST-Pgm (692-762) carrying the C712S substitution (CRD C712S) and the double mutant GST-Pgm (692-762) carrying the H701S and C712S substitutions (CRD H701S+C712S), with acid extracted histones from *P. tetraurelia* (for the control of acid extracted histone preparation and full size blots see Fig. S4). (b) Ratio of the quantified volume of bound versus input H3 bands from the *in vitro* pulldown assay. (c) ELISA assays using 100, 20 and 10 nM of GST alone or GST fused to CRD WT, CRD C712S and CRD H701S+C712S with the H3(1-19)-b peptide. (d) ELISA assays using ~100 nM of MBP alone or MBP fused to CRD WT or CRD H701S+C712S with H3(1-19)-b. MBP fusion proteins where loaded into the wells either in absence of EDTA (left) or in the presence of 10mM EDTA (right). Error bars represent the standard deviation between technical triplicates. For the control of MBP and GST fusions used in ELISA see Fig. S11.

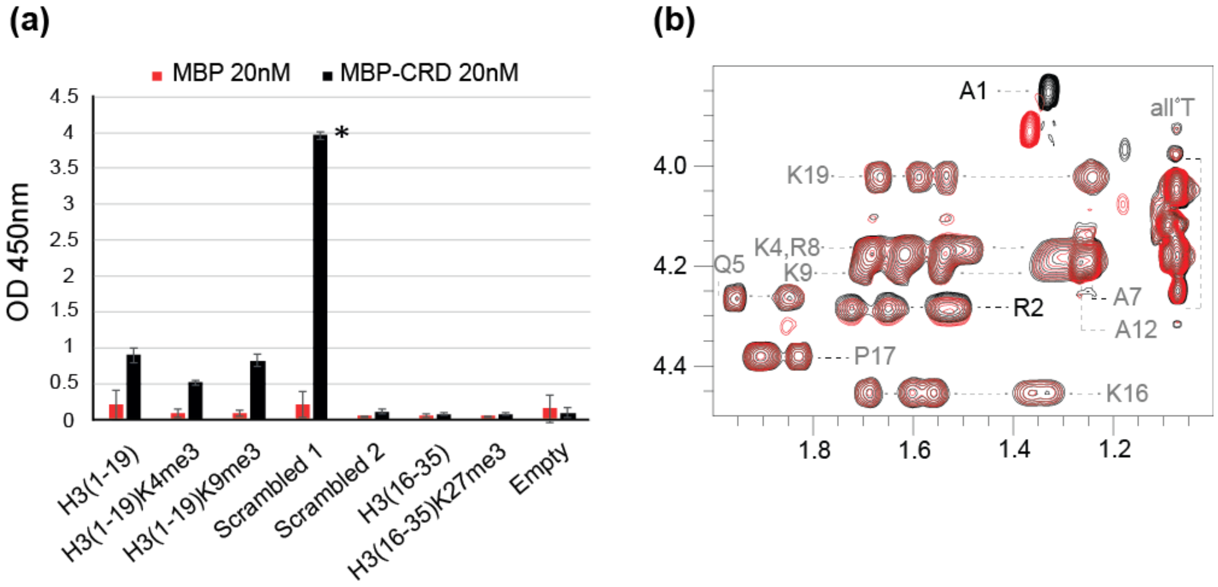

Fig. S7. Evidence for an interaction between the Pgm CRD and the N terminus of H3(1-19). (a) ELISA assays with 20 nM MBP or MBP-Pgm(692-768) (MBP-CRD) against unmodified H3(1-19)-b, trimethylated H3(1-19)K4me3-b and H3(1-19)K9m3-b, control peptides Scrambled 1 (*: TECAN absorbance detection at 450 nm close to saturation) and Scrambled 2, unmodified H3(16-35)-b and trimethylated H3(16-35)K27me3-b. Error bars represent the standard deviation between technical triplicates. (b) Selected region of the ^{1}H-^{1}H TOCSY spectrum of 1 mM H3(1-19) in the absence (in black) and presence of 0.1 mM Pgm(692-768)* (red) at pH 6.8, 20°C and 800 MHz ^{1}H. The resonances of N-terminal residues Ala1 and Arg2 are shifted by this interaction (black labels) while the resonances of other residues do not appear to be shifted (grey labels).

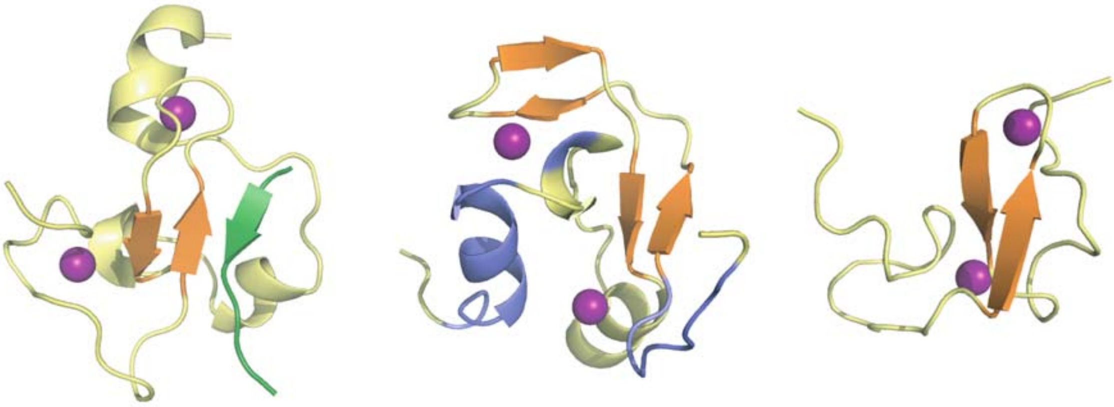

Fig. S8. Comparison of the folding of different CRDs. (a) The PHD domain of human BPTF in complex with H3(1-15)K4me3 peptide (PDB ID 3FUU) provides an example of the interaction between a PHD domain and a histone tail. The H3(1-7) N-terminal domain of histone H3 is colored in green. (b) The Pgm CRD, in which the regions contacting the H3 tail are highlighted in blue. (c) The PB CRD. In all panels, the β-sheets that may be accessible for an interaction with histone tails are colored in orange. Zn^{2+} ions are represented by purple spheres.

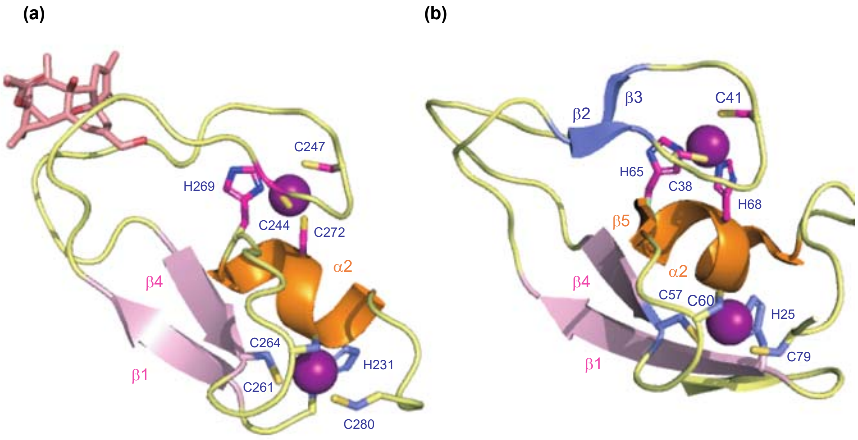

Fig. S9. Comparison of the structure of typical and atypical C1 domains. (a) Typical C1 domain of protein kinase Cδ complexed with phorbol ester (PDB id 1PTR). The phorbol ester is drawn as pink sticks. (b) Atypical C1 domain of the Rho-associated protein kinase 2 ROCK II (PDB id 2ROW). Typical and atypical C1 domains adopt similar folds but differ in their number of β strands. In contrast to the typical C1 domain, which has a single β sheet, the atypical C1 domain of ROCK II harbors two β sheets and is therefore more similar to the Pgm CRD.

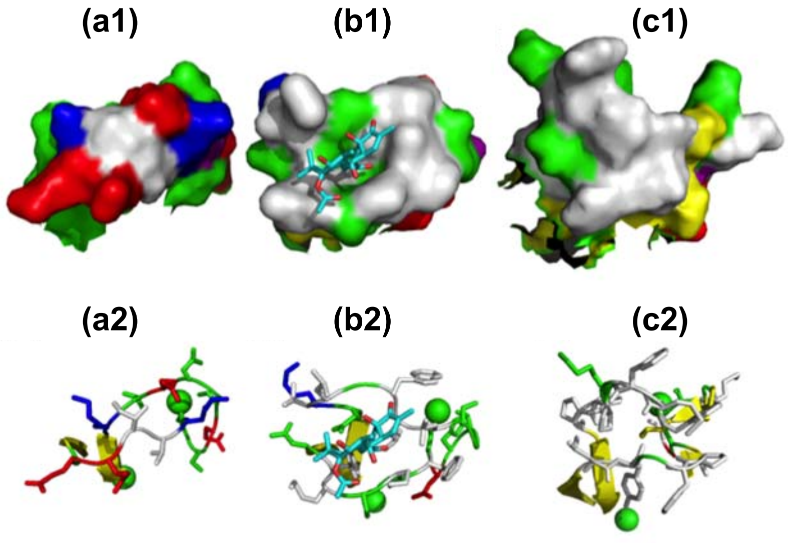

Fig. S10. Comparison of the long bent hairpin present in Pgm and in typical and atypical C1 domains. The Pgm(706-720) hairpin is displayed in a1 and a2, the typical C1 domain of the cys2 activator-binding domain of protein kinase C delta (PDB ID:1PTR) in b1 and b2 and the atypical C1 domain of the Rho kinase II (PDB ID 2ROW) in c1 and c2. Acidic residues are colored in red, basic residues in blue, hydrophobic residues in grey and all other residues are in green.

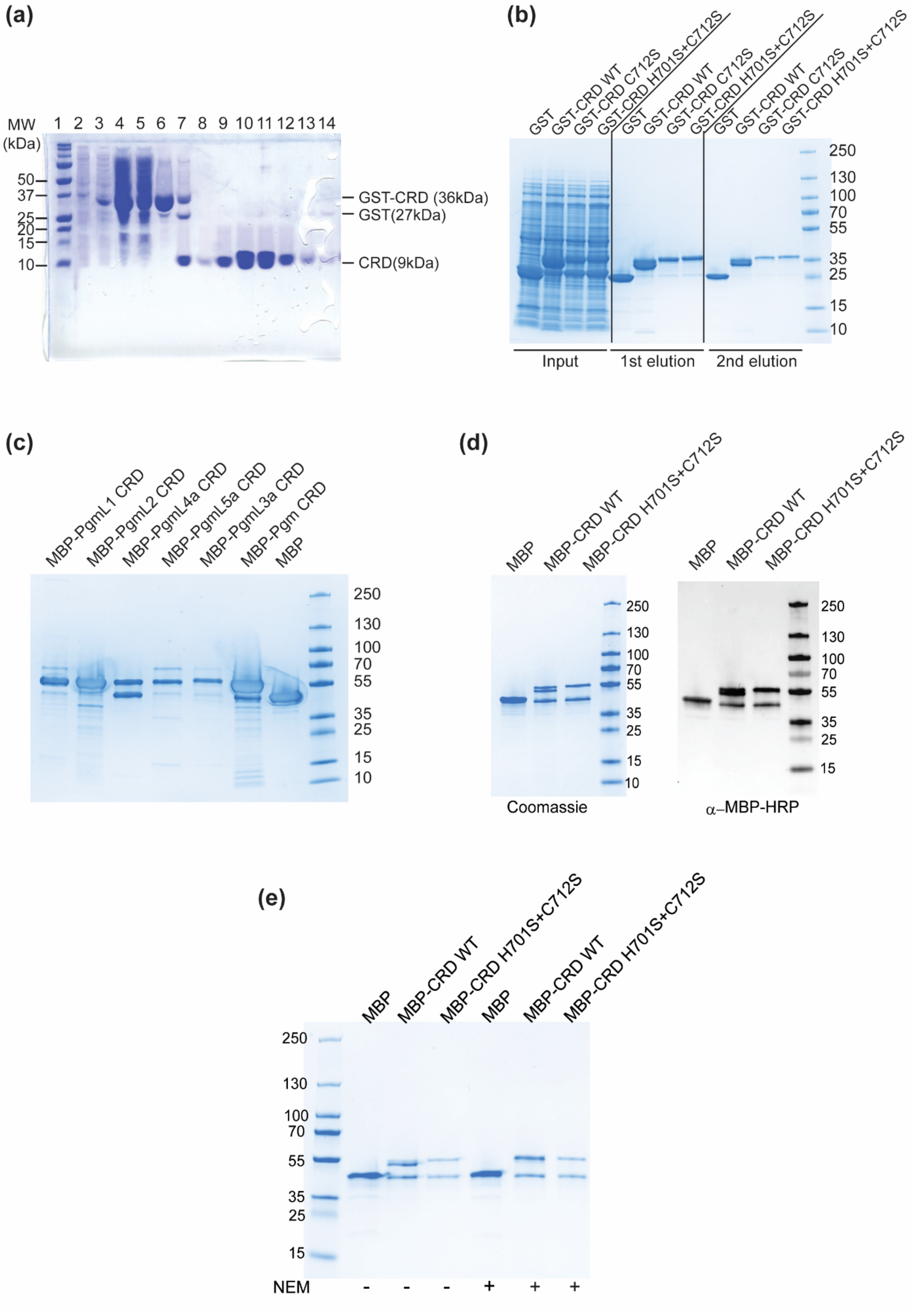
(a)
MW (kDa)
1 2 3 4 5 6 7 8 9 10 11 12 13 14
50 37 25 20 15 10
GST-CRD (36kDa)
GST(27kDa)
CRD(9kDa)
(b)
GST
GST-CRD WT
GST-CRD C712S
GST-CRD H701S+C712S
250 130 100 70 55 35 25 15 10
Input
1st elution
2nd elution
(c)
MBP-PgmL1 CRD
MBP-PgmL2 CRD
MBP-PgmL4a CRD
MBP-PgmL5a CRD
MBP-PgmL3a CRD
MBP-Pgm CRD
MBP
250 130 100 70 55 35 25 15 10
(d)
MBP
MBP-CRD WT
MBP-CRD H701S+C712S
250 130 100 70 55 35 25 15 10
Coomassie
α–MBP-HRP
(e)
MBP
MBP-CRD WT
MBP-CRD H701S+C712S
250 130 100 70 55 35 25 15
NEM - - - + + +

Fig. S11. Expression and purification of the wild-type and mutant CRDs used in this study. (a) 15% SDS-PAGE gel summarizing the purification steps used to produce the wild-type Pgm(692-768)* peptide for NMR experiments. 1: molecular weight marker; 2: total cell extract before induction with 0.2 mM IPTG; 3: total cell extract after IPTG induction; 4: clear supernatant; 5: glutathione Sepharose beads, unbound fraction; 6: glutathione Sepharose beads, bound fraction; 7: PreScission cleavage supernatant; 8-14: elution fractions from Hitrap Q HP. (b) Expression and purification of wild-type and mutant GST-Pgm(692-768) for DNA and histone binding assays. (c) Purified preparations of MBP-PgmL1(463-539), MBP-PgmL2(540-614), MBP-PgmL3a(471-550), MBP-PgmL4a(856-931), MBP-PgmL5a(761-840), MBP-Pgm(692-768) and MBP used for DNA and histone binding assays. (d) Purified preparations of MBP alone, MBP-Pgm(692-768) (MBP-CRD WT) and MBP-Pgm(692-768) double mutant (MBP-CRD H701S+C712S) used for ELISA assays. For those preparations, in which some of the free MBP tag was released upon over-expression or during bacterial lysis, only the full-length fusion protein was taken into account to calculate the amount of input CRD in histone-binding assays. (e) Effect of alkylation agent N-ethyl-maleimide (NEM) on intramolecular S-S bond formation in the wildtype Pgm CRD. MBP, MBP-CRD WT or MBP-CRD H701S+C712S were preincubated for 10 min with (+) or without (-) 20 μM NEM before denaturation (boiling in 1X Laemmli buffer + 357 mM β-mercaptoethanol) and loading on an SDS-PAGE gel. NEM-induced disappearance of the lower band of the MBP-CRD WT doublet indicates that the doublet (observed in panels b, d and e) results from intramolecular disulfide bond formation within the wildtype Pgm CRD. Panels b to e show 5-15% SDS-PAGE gels.

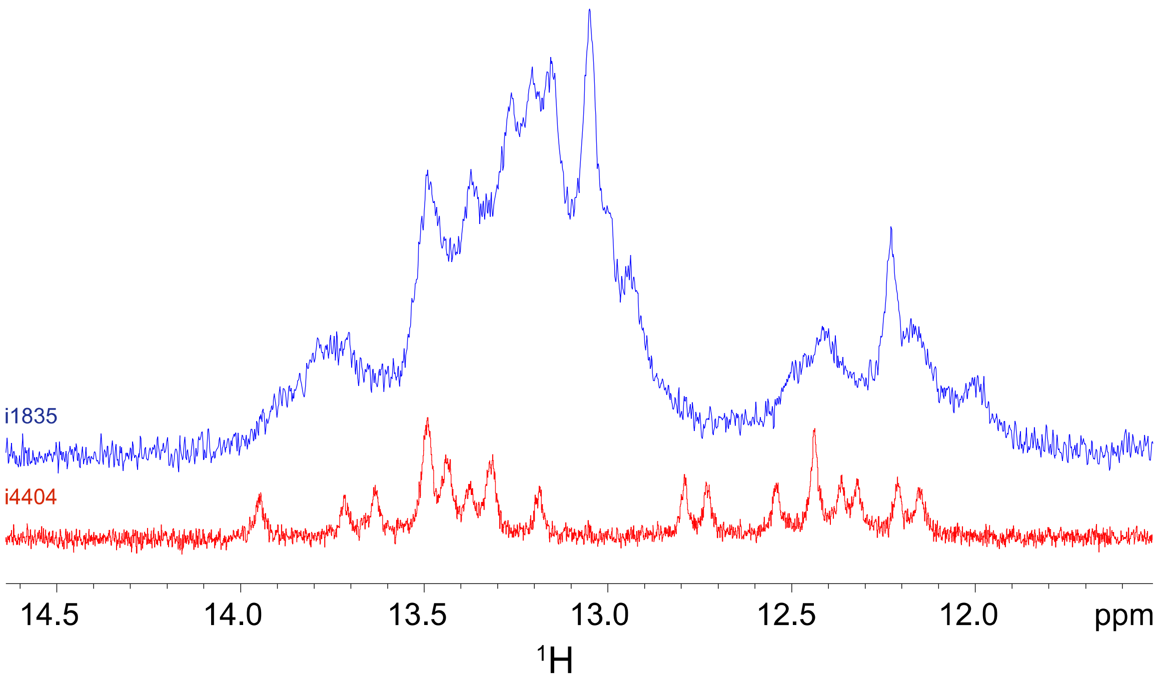

Fig. S12. ¹H-NMR spectra of imino protons of i1835 (blue) and i4404 (red). Their presence shows that DNA was correctly annealed prior to interaction studies. i1835: CTACTACATAATGCTAAACTCATTTATAGATGGATTGTTTTCCAAGTATCTATATC and its complementary strand; i4404: GCTTGCACATCTCTAGTTGATGG and its complementary strand (see Fig. S4b).

SUPPLEMENTARY TABLES

Table S1. Comparison of zinc-binding capability of histidine mutants of Pgm(692-768). Microwave-plasma atomic emission spectroscopy was used to determine the amount of zinc in wild-type (wt) and histidine-to-serine mutants of GST-fused Pgm(692-768). Zinc concentrations were determined both with and without a washing step with 2 mM EDTA.

GST-Pgm(692-768)	No EDTA*	2 mM EDTA#
Wt+	1	1
H701S	0.42 ± 0.06	0.38 ± 0.01
H738S	1.32 ± 0.13	1.18 ± 0.01
C712S	0.23 ± 0.04	0.16 ± 0.01

+ Normalization of zinc concentration for the mutants by that measured for Pgm Wt.

* Average on two measurements recorded for both the Wt and the three mutants

# Calculated using the average of two measurements recorded for the Wt. Only one measurement was recorded for each mutant. 2 mM EDTA was used to complex the possible excess zinc in the solutions. We verified by NMR that 2 mM of EDTA is not strong enough to compete with the zinc binding sites of Pgm(692-768).

The impact of a mutation on the overall structure of the CRD and its ability to bind zinc ions may very strongly depend on the position of the mutated Cys or His residue. We observe that the Cys712S mutation has a greater impact on the zinc-binding ability of the CRD than the H701S mutation. Thus, the mutation of Cys712, which participated in the formation of the 2nd zinc-coordinating motif (and is located further inside the sequence, see Fig. 3), probably has a greater impact on the overall structure of the CRD than the H701S mutation, which affects only the 1st ligand of the first zinc-ccordinating motif.
